# *β*-Cyclodextrin Catalyzed, One-Pot Multicomponent Synthesis and Antimicrobial Potential of N-Aminopolyhydroquinoline Derivatives

**DOI:** 10.3390/molecules29194655

**Published:** 2024-09-30

**Authors:** Sonali Garg, Manvinder Kaur, Pradip K. Bhowmik, Harvinder Singh Sohal, Fohad Mabood Husain, Haesook Han

**Affiliations:** 1 Medicinal and Natural Product Laboratory, Department of Chemistry, Chandigarh University, Gharuan, Mohali 140413, Punjab, India; 2 Department of Chemistry and Biochemistry, University of Nevada Las Vegas, 4505 S. Maryland Parkway, Box 454003, Las Vegas, NV 89154, USA; 3 Department of Food Science and Nutrition, College of Food and Agriculture Sciences, King Saud University, Riyadh 11451, Saudi Arabia

**Keywords:** polyhydroquinoline, *β*-cyclodextrin, catalyst, Hantzsch reaction, antimicrobial activity

## Abstract

In the present report, we have described the synthesis of N-aminopolyhydroquinoline (N-PHQ) derivatives using highly efficient *β*-cyclodextrin (*β*-CD) as a catalyst by the Hantzsch condensation of substituted aromatic aldehydes, dimedone, and hydrazine hydrate in one pot. The reactions were completed in a shorter time without the generation of any other byproduct. The synthesized N-PHQs were washed thoroughly with distilled water and recrystallized with ethanol to get highly purified products (as crystals). The structure of the synthesized N-PHQs was established by using advanced spectroscopic techniques *like* FT-IR, NMR (^1^H, ^13^C, DEPT, COSY, and HSQC), ESI-MS, and Elemental Analyzer. The N-PHQs derivatives demonstrated moderate to excellent resistance against the tested strains (both fungal as well as bacterial). The presence of polar groups, which are able to form H-bonds, attached to the phenyl ring *like* -NO_2_ (**4b** and **4c)**, and -OMe (**4i, 4j,** and **4k**) exhibits excellent activity, which is comparable to standard drugs, amoxicillin and fluconazole.

## 1. Introduction

Polyhydroquinoline (PHQ) derivatives belong to a significant group of heterocyclic compounds that have emerged as flexible biologically active compounds with various applications, such as anti-tumor [1], anti-atherosclerotic [2], bronchodilator [3], antidiabetic activity [4], and have the ability to modulate calcium channels [5], diuretics [6], vasodilators [7], tranquilizers [8], antibiotic [5,9], anti-defibrillatory [5], anticonvulsant [10], antihistaminic [11], herbicidal activity [12], plant growth regulation ability [13], anticancer [14,15], antihypertensive agents [16], and chemosensitizer in tumor therapy [3].

The synthesis of N-PHQ derivatives prominently features the Hantzsch reaction, a classical one-pot, multi-component reaction involving an aldehyde, *β*-ketoester, ammonium acetate, and active methylene compound. Despite its efficiency and ability to yield diverse N-PHQs, it often requires long reaction times and high temperatures. To improve this, microwave-assisted synthesis reduces reaction times and increases yields while enhancing simplicity and energy efficiency [17,18]. Green chemistry approaches, *like* using ionic liquids [19], TMSCl-NaI [20], HY-Zeolites [21], ceric ammonium nitrate (CAN) [22], *p*-TSA [10], polyacids [23], L-proline [24], ZrCl_4_ [25], Yb(OTf)_3_ [26], TiO_2_ and Fe_3_O_4_ nanoparticles [27], and Mont. K-10 [28]. The approaches to carry this reaction include conventional heating [29,30], microwave irradiation [31], and ultrasonic irradiation [32], among which microwave (MW) [33] and UV irradiation [34] are demonstrated to be more efficient and time-saving. Catalysis, employing Lewis’s acids [35], Brønsted acids [19,36], and metal-based catalysts, further enhances efficiency and selectivity, with notable success using lanthanide triflates and transition metals [26].

In the realm of green chemistry, the design and development of organic synthesis through multicomponent reactions (MCRs) have emerged as a critical research focus in organic chemistry. This approach aligns with the principles of sustainable chemistry, promoting environmentally friendly practices and resource conservation [36]. Therefore, MCRs often serve as efficient alternatives to traditional sequential multistep synthesis. The synergistic combination of *β*-CD with MCR enhances the synthesis of diverse nitrogen-containing heterocycles. One prominent method for preparing these compounds is the Hantzsch condensation reaction, which produces polyhydroquinolines [37]. By using *β*-CD as the catalyst, this approach improves reaction efficiency. The Hantzsch condensation is particularly valued for its ability to generate a wide variety of N-PHQ, compounds that are significant in pharmaceutical and materials science due to their diverse biological activities and functional properties [38].

*β*-CD, a cyclic oligosaccharide, possesses a unique molecular structure with a hydrophobic cavity. This cavity allows it to encapsulate hydrophobic guest molecules, including various organic compounds. Researchers have explored *β*-CD’s antimicrobial properties due to its ability to form inclusion complexes with lipophilic substances [39]. By trapping these substances, *β*-CD can inhibit microbial growth. Instead of solely investigating *β*-CD’s antimicrobial properties, we harnessed *β*-CD as a catalyst in the synthesis of N-aminopolyhydroquinoline derivatives. These derivatives hold immense promise in various applications, such as pharmaceuticals, dyes, and antioxidants. By using *β*-CD as a catalyst, we aimed to achieve efficient and selective transformations, leveraging its unique cavity for molecular interactions [40].

Therefore, the development of a clean and efficient synthesis method for N-PHQ derivatives is still timely. Understanding the importance of both N-PHQs and MCRs, in the present report, a novel and green protocol has been presented, which is much more efficient than existing procedures.

## 2. Result and Discussion

### 2.1. Optimization of Reaction Conditions

#### 2.1.1. Effect of Catalyst and Solvent

**Scheme 1:**
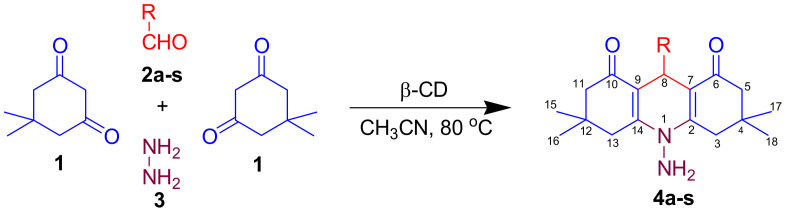
Synthesis of N-PHQ derivatives **4a–s** using *β*-CD as a catalyst.

The reaction conditions were standardized by numerous combinations of catalysts (5 mmol%) with solvent systems, which were examined on model reaction. The model reaction (Scheme 1) involved the condensation of 5,5-dimethylcyclohexane-1,3-dione **1** (2 eq.), benzaldehyde **2a** (1 eq.), and hydrazine hydrate **3** (1 eq.) refluxed at 80 °C in the presence of various combinations of catalyst (5 mmol%) and solvent (5 mL) system to yield *10-amino-3,3,6,6-tetramethyl-9-phenyl-3,4,6,7-tetrahydroacridine-1,8(2H,5H,9H,10H)-dione* **4a**. We confirmed the reaction progress using TLC (hexane: ethyl acetate/8:2) and validated the product by comparing its melting point to literature data.

Initially, to get the product, the reaction was carried out without a catalyst with various solvents (ethanol, acetonitrile (ACN), and diethyl ether). To improve the reaction yields, a small quantity of catalyst, i.e., piperidine, was added to the reaction mixture, along with the above-mentioned solvents. Thereafter, the reaction was carried out in water using sodium dodecyl sulfate, Al(DS)_3_, Sc(DS)_3_, and 2-aminopyrazine as a catalyst. Among all, 2-aminopyrazine was found to be an inefficient catalyst, and no desired product was formed. However, using *β*-CD as a catalyst in ACN, ethanol, and diethyl ether not only improves the yield of the reaction but reduces the time of the reaction as well. Out of the various adopted methodologies, a combination of *β*-CD in ACN (Table 1) turns out to be the superlative catalyst–solvent combination for the synthesis of compound **4a** in very high yield and purity (Table 1). The products were then purified by a simple recrystallization procedure.

#### 2.1.2. Effect of Temperature

After optimization of the catalyst–solvent combination, the model reaction was further examined under different temperature ranges (50–95 °C) (Table 2). It was observed that, at lower temperatures, the yield of the produced product was very low, while, by increasing the temperature, the enhancement in yield was observed up to 96%. Conversely, elevating the temperature beyond temperature 80 °C led to a decline in product yield, attributed to the decomposition of products at higher temperatures. The results obtained using this methodology are given in Scheme 1.

Similarly, different aldehydes were reacted with dimedone and hydrazine hydrate using the same procedure, and their formation was monitored (Table 3). From Scheme 1, it was observed that the electron-withdrawing group (EWG) **4a–h** substituent on the aromatic ring speeds up the reaction. A higher yield was obtained in a short period, while the electron-donating group (EDG) slowed down the rate of reaction.

### 2.2. Chemistry

**Figure 1:**
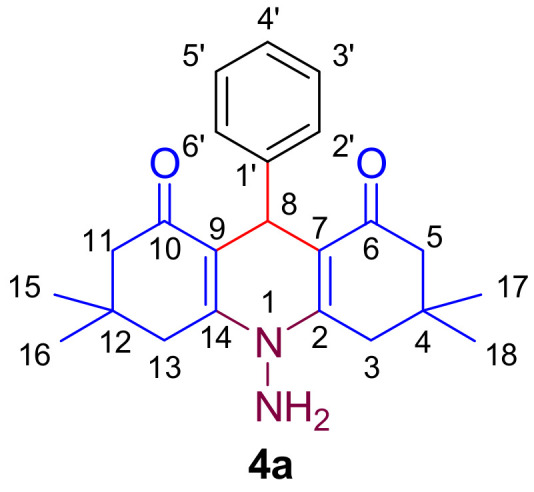
Structure of 10-amino-3,3,6,6-tetramethyl-9-phenyl-3,4,6,7-tetrahydroacridine-1,8(2H,5H,9H,10 H)-dione (**4a**).

The various spectroscopic techniques, i.e., FT-IR spectroscopy (with diamond ATR), NMR (^1^H, ^13^C, and DEPT NMR), 2D NMR (COSY and HSQC), and mass spectroscopy were used to confirm the structure of the synthesized compounds (**4a**–**s**). The chemical structure of compound **4a** is given in Figure 1.

The functional group of the compounds was confirmed by IR spectra; the characteristic stretching peaks of compound **4a** were observed at 3326.77 cm^−1^ for NH_2_, 3046 cm^−1^ for *sp^2^* C-H bonds, 2959 and 2929 cm^−1^ for *sp^3^* C-H bond, and 1615.16 cm^−1^ for C=O bond.

In ^1^H NMR spectra (500 MHz, DMSO) of compound **4a**, triplet at *δ* 7.14–7.13 ppm and multiplet at *δ* 7.05–7.02 ppm was observed for protons of phenyl ring at C-4 position. Two singlets at *δ* 5.28 and *δ* 4.95 ppm were observed for the NH_2_ group and H-4 groups, respectively, whereas peaks of CH_2_ groups present in N-PHQs were observed at *δ* 2.91–2.87 (2H, H-11), *δ* 2.54 (2H, H-5), *δ* 2.17–2.14 (2H, H-13), and *δ* 2.02–1.99 (2H, H-3) ppm. The two singlet peaks of four methyl groups were observed at *δ* 1.02 (6H, 2*CH_3_) and *δ* 0.86 (6H, 2*CH_3_) ppm, respectively.

In ^13^C NMR spectra (125 MHz, DMSO) of molecule **4a**, *δ* 194.44 ppm was observed for the C=O group, which was the most down-shielded peak. Peaks at *δ* 154.47 and *δ* 146.33 ppm were observed for C-2 and C-14 and C-7 and C-9 carbon atoms, whereas carbon belonging to the aromatic part was observed at *δ* 127.49, 127.20, and 125.38 ppm. The peaks for *sp^3^* carbons were observed at 31.24 (C-8), 49.51 (C-3 and C-13), 38.50 (C-5 and C-11), 29.38 (CH_3_), and 26.57 (CH_3_) ppm, respectively. Further, these carbon peaks were confirmed by ^13^C DEPT 135.

Further to determine H-H coupling and C-H coupling, 2D NMR spectra COSY and HSQC, respectively, were taken. HSQC confirms that the aromatic carbon peaks observed at *δ* 127.49, and 127.20 couple with hydrogen at *δ* 7.14–7.13 (4H), and *δ* 125.38, couples with hydrogen at *δ* 7.05–7.02 (1H). These peaks remain sharp, suggesting that the aromatic part of the N-PHQ may not be deeply involved in the inclusion complex formation, or it may reside near the exterior of *β*-CD, not fully inside the hydrophobic cavity [43]. The CH_2_ peaks at 49.51 (C-3, C-13), and 38.50 (C-5, C-11) couples with hydrogen at *δ* 2.91–2.87 (2H, H-11), *δ* 2.54 (2H, H-5), *δ* 2.17–2.14 (2H, H-13), and *δ* 2.02–1.99 (2H, H-3), respectively. These aliphatic groups, particularly CH_2_ groups, may interact with the inner hydrophobic cavity of *β*-CD since it preferentially hosts nonpolar regions of guest molecules inside its cavity. The C-H (*sp^3^*) peak at 31.24 coupling with singlet at *δ* 4.95 is crucial because *sp^3^* carbons are more likely to fit into the hydrophobic cavity of *β*-CD. The chemical shift changes observed here (*sp^3^* carbons coupling with protons) may suggest that this region is involved in the inclusion process. The four CH_3_ of dimedone moiety gave carbon peaks at 29.37 and 26.57, which couples with hydrogen *δ* 1.0276 (6H, 2*CH_3_) and *δ* 0.8603 (6H, 2*CH_3_), respectively. These groups are relatively small and hydrophobic, making them ideal candidates to fit inside the *β*-CD cavity. Furthermore, COSY, EI-MS, and elemental analysis verified the synthesis of the desired compound.

### 2.3. Plausible Mechanism

**Scheme 2:**
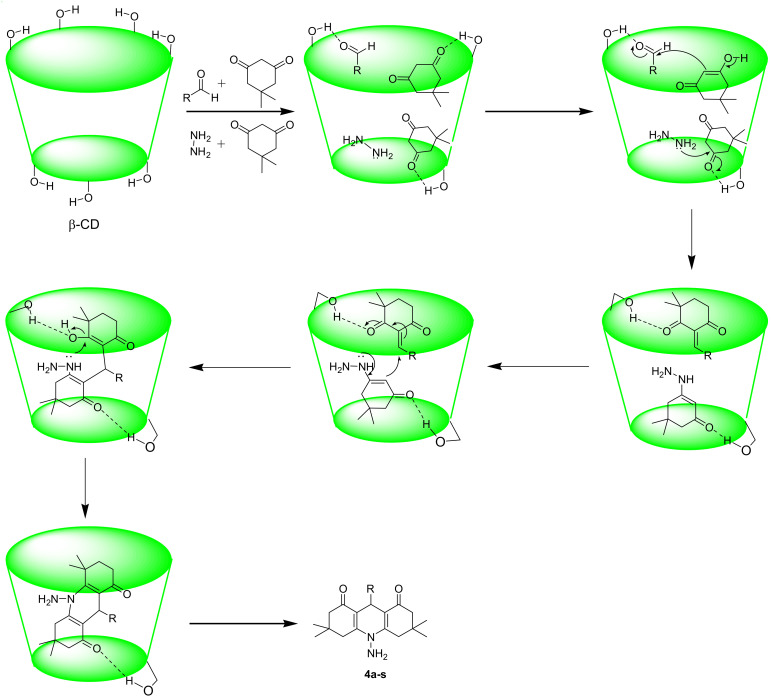
A plausible mechanism for the synthesis of N-PHQ derivatives **4a–s** using *β*-CD as a catalyst.

The reasonable mechanism for the synthesis of N-PHQs is described in Scheme 2. The initial step proceeds with Knoevenagel condensation between aldehyde and dimedone to produce an enone system in the cavity of *β*-CD, and parallel in the same cavity, hydrazine undergoes a condensation reaction with the other molecule of dimedone, followed by the removal of water to form an enamine. Further, both the intermediate products were activated in the *β*-CD cavity by the formation of H-bonding and underwent Michael addition, followed by the removal of water to generate a final cyclic product. In the final step, the expulsion of final products (**4a**–**s**) takes place from the cavity of *β*-CD.

The primary interaction between *β*-CD and N-PHQ derivatives occurs through the formation of inclusion complexes [44]. In this process, the hydrophobic moiety of the N-PHQ derivatives enters the hydrophobic cavity of *β*-CD, while the polar groups remain exposed to the aqueous environment, and this encapsulation stabilizes the N-PHQ derivatives.

The inclusion complex is stabilized by non-covalent interactions, such as hydrogen bonding, van der Waals forces, and hydrophobic interactions. Hydrogen bonds can form between the hydroxyl groups on the rim of *β*-CD and the functional groups (e.g., amines, carbonyls) on the N-PHQ derivatives, enhancing the strength and specificity of the complex. If the N-PHQ derivatives contain aromatic rings, π-π stacking interactions between the aromatic groups of the guest molecule and the glucose units of *β*-CD may also contribute to the stabilization of the complex [45].

### 2.4. Antimicrobial Evaluation

The minimum inhibitory concentration (MIC) method was employed to verify the antimicrobial characteristics of the synthesized compounds **4a–s**. The MIC standards of the compounds were compared to those of the mentioned drugs, amoxicillin (4 μg/mL) and fluconazole (2 μg/mL), to assess their effectiveness. From Table 4, it is evident that compounds of series **4a**–**s** show great interaction with numeral microbes (both fungal and bacterial). Among the entire tested compounds, only those compounds that have polar groups attached to the phenyl ring (**4b**, **4c**, **4i**, **4j**, and **4k**) were deemed to be more efficient against all the tested strains because they can form hydrogen bonding with the microbial’s protein. Compound **4k** having two polar groups showed maximum activity among all the compounds, which is quite *like* the reference drug, amoxicillin, at MIC 4 μg/mL. It has also been observed that if the phenyl group were replaced by some alkyl group (**4r** and at MIC 4 μg/mL), the compounds become very less effective (MIC 64 and 128 μg/mL) against these strains.

The structure-activity relationship (SAR) in this series of compounds shows a clear correlation between the electronic nature of the substituents and their antimicrobial efficacy. In general, EWGs, such as nitro (-NO₂), chloro (-Cl), and bromo (-Br), tend to enhance antimicrobial activity in specific cases. For example, compound **4b** (4-NO₂) demonstrates improved activity against Gram-positive bacteria, *B. subtilis*, and *S. pyogenes*, with MIC values of 8 and 4 μg/mL, respectively, compared to compound **4a**, which lacks a strong EWG. Similarly, compound **4c** (3-NO₂) displays potent activity across most tested strains, particularly *E. coli*, *K. pneumoniae*, and various fungi. The ability of the nitro group to withdraw electron density likely contributes to better binding interactions with microbial targets, enhancing overall efficacy [46].

On the other hand, EDGs, such as methoxy (-OCH₃) and methyl (-CH₃), appear to have mixed effects on antimicrobial activity. Compound **4k**, with two methoxy groups (3,4-(OCH₃)₂), exhibits consistently strong activity against both Gram-positive and Gram-negative bacteria, with MIC values of 4 μg/mL across all tested strains. This suggests that certain EDGs can still promote favorable interactions with microbial enzymes or cell membranes. However, compounds with single methoxy or methyl groups, such as **4i** (4-OCH₃) and **4l** (4-CH₃), show moderate to lower activity compared to their electron-withdrawing counterparts, indicating that EDGs might not uniformly enhance antimicrobial properties [47].

The positional effects of these substituents also play a crucial role in determining activity. For instance, compound **4f** (2-Cl) shows significantly reduced activity compared to **4e** (4-Cl), with MIC values up to 64 μg/mL against Gram-positive bacteria. This highlights the importance of both the electronic nature and the spatial orientation of substituents in optimizing antimicrobial efficacy. Overall, the introduction of EWGs, particularly in para and meta positions, appears to strengthen antimicrobial activity, whereas EDGs require specific positioning to maintain or enhance biological effects.

### 2.5. Comparison of the Catalytical Activity β-CD with Previous Literature Reports for the Synthesis of ***4a***

Table 5 presents a comparison between previous studies and the current work. It clearly shows that the current research employs significantly milder reaction temperatures, unlike other reported methods that require higher temperatures or reflux conditions. (See entries 1–3 in Table 5) This highlights the improved efficiency of the present method under more favorable reaction conditions. Moreover, some earlier reports involve time-some procedures for preparing the catalyst (see Table 5, entry 3), whereas preparing *β*-CD is simpler. Consequently, this study offers notable benefits, including the use of a straightforward catalyst, eco-friendliness, milder conditions, and improvements over previous methods for synthesizing N-PHQ derivatives.

## 3. Materials and Methods

The entire chemicals utilized in this research were procured from Sigma Aldrich (Mumbai, India) and were used without any additional refining, while the solvents were prearranged from Loba Chemie (Ludhiana, India). The melting point apparatus (digital) was used to measure the melting point via the open capillary method for all the synthesized products. IR spectra of the targeted compound were taken using ATR mode on Perkin Elmer Spectrum II (Chandigarh University, Gharuan, Punjab, India). NMR, such as ^1^H, ^13^C, DEPT, HSQC, and COSY, were collected using DMSO as a solvent on a Bruker Avance NEO 500 MHz NMR spectrometer (Punjab University, Chandigarh, India). Chemical shifts (δ) were accounted for in ppm relative to that of TMS as an internal standard. The mass spectroscopy was analyzed on LC-MS Spectrometer Model Q-ToF Micromass. For fundamental analysis, Thermo Scientific (FLASH 2000) CHN Elemental Analyzer was used (Punjab University, Chandigarh, India). The thin layer chromatographic (TLC) technique was used to observe the reaction time as well as to check the purity of the compound, and then with the help of a UV chamber, the visualization of TLC was done.

## 4. Experimental

### 4.1. Characterization of Synthesized Polyhydroquinolines Derivatives (***4a***–***s***)

***10-amino-3,3,6,6-tetramethyl-9-phenyl-3,4,6,7-tetrahydroacridine-1,8(2H,5H,9H,10H)-dione (*4a*)*:** Yield 96%, pale yellow crystalline solid, m.p. 260–261 °C. IR Spectrum (υ, cm^−1^): 3326.77 (N-H), 3046.1 (C=C-H), 2959.01, 2929.2 (*sp^3^* C-H), and 1615.16 (C=O). ^1^H NMR spectrum (DMSO, 500 MHz): 7.14–7.13 (t, 4H, H-2′, H-3′ H-5′, H-6′), 7.05–7.0250 (m, 1H, H-4′), 5.28 (s, 2H, N-H_2_), 4.95 (s, 1H, H-8), 2.91-1.99 (*m*, 8H, 2*H-3, 2*H-5, 2*H-11, 2*H-13), 1.02 (s, 6H, 2*CH_3_), 0.86 (s, 6H, 2*CH_3_). ^13^C NMR spectrum (DMSO, 125 MHz): 194.44 (C=O), 154.47 (C-2, C-14), 146.33 (C-7, C-9), 127.49, 127.20, 125.38 (Ph), 110.95 (C-4, C-12), 49.51 (C-3, C-13), 38.50 (C-5, C-11), 31.24 (C-8), 29.37 (CH_3_), 26.57 (CH_3_). Anal. calcd. for C_23_H_28_N_2_O_2_: C, 75.79; H, 7.74; N, 7.69. Found; C, 75.5 6 H, 7.93; N, 7.56%. ESI-MS (*m/z*); M + 1 = 365.62.
***10-amino-3,3,6,6-tetramethyl-9-(4-nitrophenyl)-3,4,6,7-tetrahydroacridine-1,8(2H,5H,9H,10H)-dione* *(*4b*)*:** Yield 95%, pale yellow crystalline solid, m.p. 270–272 °C. IR Spectrum (υ, cm^−1^): 3426.77 (N-H), 3146.1 (C=C-H), 2989, 2959 (*sp^3^* C-H), and 1665.16 (C=O), 1510, 1342 (NO_2_). ^1^H NMR spectrum (CDCl_3_, 500 MHz): 8.11-8.14 (d, 2H, H-2′, H-6′), 7.24–7.28 (d, 2H, H-3′, H-5′), 5.34 (s, 2H, NH_2_), 5.01 (s, 1H, H-8), 2.32-2.51 (*m*, 8H, 2*H-3, 2*H-5, 2*H-11, 2*H-13), 1.23 (s, 6H, 2*CH_3_), 1.11 (s, 6H, 2*CH_3_). ^13^C NMR spectrum (CDCl_3_, 125 MHz): 190.90 (C=O), 146.56 (C-2, C-14), 146.08 (C-7, C-9), 127.63, 123.47 (Ph), 114.87 (C-4, C-12), 46.96 (C-3, C-13), 33.23 (C-5, C-11), 31.45 (C-8), 29.46 (CH_3_), 27.44 (CH_3_). Anal. calcd. for C_23_H_27_N_3_O_4_: C, 67.46%; H, 6.65%; N, 10.26%. Found: C, 67.65%; H, 6.25%; N, 10.38%. ESI-MS (*m/z*); M + 1 = 410.20.
***10-amino-3,3,6,6-tetramethyl-9-(3-nitrophenyl)-3,4,6,7-tetrahydroacridine-1,8(2H,5H,9H,10H)-dione (*4c*)*:** Yield 92%, pale yellow crystalline solid, m.p. 223–225 °C. IR Spectrum (υ, cm^−1^): 3356.75 (N-H), 3206.1 (C=C-H), 2979, 2945 (*sp^3^* C-H), and 1635.16 (C=O), 1525, 1346 (NO_2_). ^1^H NMR spectrum (CDCl_3_, 500 MHz): 7.14 (d, 1H, H-6′), 7.13 (s, 1H, H-2′), 7.05 (t, 1H, H-5′), 7.02 (d, 1H, H-4′), 5.38 (s, 2H, NH_2_), 5.54 (s, 1H, H-8), 2.92–2.32 (*m*, 8H, 2*H-3, 2*H-5, 2*H-11, 2*H-13), 1.12 (s, 6H, 2*CH_3_), 1.07 (s, 6H, 2*CH_3_). ^13^C NMR spectrum (CDCl_3_, 125 MHz): 191.06 (C=O), 148.40 (C-2, C-14), 140.71 (C-7, C-9), 132.87, 129.07, 122.20, 121.00 (Ph), 114.74 (C-4, C-12), 46.99 (C-3, C-13), 32.86 (C-5, C-11), 31.41 (C-8), 29.64 (CH_3_), 27.26 (CH_3_). Anal. calcd. for C_23_H_27_N_3_O_4_: C, 67.46%; H, 6.65%; N, 10.26%. Found: C, 67.65%; H, 6.25%; N, 10.38%. ESI-MS (*m/z*); M + 1 = 410.20.
***10-amino-3,3,6,6-tetramethyl-9-(2-nitrophenyl)-3,4,6,7-tetrahydroacridine-1,8(2H,5H,9H,10H)-dione (*4d*)*:** Yield 89%, pale yellow crystalline solid, m.p. 210–212 °C. IR Spectrum (υ, cm^−1^): 3453.75 (N-H), 3215.1 (C=C-H), 2981, 2930 (*sp^3^* C-H), and 1629.16 (C=O), 1530, 1349 (NO_2_). ^1^H NMR spectrum (CDCl_3_, 500 MHz): 7.29–7.23 (d, 1H, H-6′), 7.33 (t, 1H, H-4′), 7.21 (d, 1H, H-3′), 7.07 (t, 1H, H-5′), 5.76 (s, 2H, NH_2_), 4.47 (s, 1H, H-8), 2.67–2.29 (*m*, 8H, 2*H-3, 2*H-5, 2*H-11, 2*H-13), 1.05 (s, 6H, 2*CH_3_), 1.01 (s, 6H, 2*CH_3_). ^13^C NMR spectrum (CDCl_3_, 125 MHz): 197.42 (C=O), 157.40 (C-2, C-14), 153.82 (C-7, C-9), 134.90, 134.01, 133.70 (Ph), 114.73 (C-4, C-12), 51.53 (C-3, C-13), 47.70 (C-5, C-11), 36.10 (C-8), 32.20 (CH_3_), 27.90 (CH_3_). Anal. calcd. for C_23_H_27_N_3_O_4_: C, 67.46%; H, 6.65%; N, 10.26%. Found: C, 67.46%; H, 6.65%; N, 10.26%, O = 15.63; ESI-MS (*m/z*); M + 1 = 410.20.
***10-amino-9-(4-chlorophenyl)-3,3,6,6-tetramethyl-3,4,6,7-tetrahydroacridine-1,8(2H,5H,9H,10H)-dione (*4e*)*:** Yield 94%, pale yellow crystalline solid, m.p. 274–275 °C. IR Spectrum (υ, cm^−1^): 3216.77 (N-H), 2982.1 (C=C-H), 2919, 2887 (*sp^3^* C-H), and 1505.16 (C=O). ^1^H NMR spectrum (CDCl_3_, 500 MHz): 7.04–7.03 (d, 2H, H-2′, H-6′), 7.01–7.00 (d, 2H, H-3′, H-5′), 5.02 (s, 2H, NH_2_), 4.96 (s, 1H, H-8), 2.91–1.98 (*m*, 8H, 2*H-3, 2*H-5, 2*H-11, 2*H-13), 1.02 (s, 6H, 2*CH_3_), 0.85 (s, 6H, 2*CH_3_). ^13^C NMR spectrum (CDCl_3_, 125 MHz): 192.44 (C=O), 151.47(C-2, C-14), 142.13 (C-7, C-9), 127.89, 126.12, 124.25 (Ph), 115.57 (C-4, C-12), 49.37 (C-3, C-13), 38.50 (C-5, C-11), 30.12 (C-8), 29.12 (CH_3_), 26.27 (CH_3_). Anal. calcd. for C_23_H_27_ClN_2_O_2_: C, 69.25; H, 6.82; N, 7.02%. Found; C, 69.15; H, 6.92; N, 7.23%. ESI-MS (*m/z*); M + 1 = 399.18, M + 2 = 400.17.
***10-amino-9-(2-chlorophenyl)-3,3,6,6-tetramethyl-3,4,6,7-tetrahydroacridine-1,8(2H,5H,9H,10H)-dione* *(*4f*)*:** Yield 91%, pale yellow crystalline solid, m.p. 203–204 °C. IR Spectrum (υ, cm^−1^): 3216.77 (N-H), 2982.1 (C=C-H), 2909, 2865 (*sp^3^* C-H), and 1505.16 (C=O). ^1^H NMR spectrum (CDCl_3_, 500 MHz): 6.99–7.26 (d, 4H, H-3′,4′,5′,6′), 5.18 (s, 2H, NH_2_), 4.94 (s, 1H, H-8), 2.61–2.32 (*m*, 8H, 2*H-3, 2*H-5, 2*H-11, 2*H-13), 1.03 (s, 6H, 2*CH_3_), 0.99 (s, 6H, 2*CH_3_). ^13^C NMR spectrum (CDCl_3_, 125 MHz): 200.89 (C=O), 169.14 (C-2, C-14), 151.04 (C-7, C-9), 127.98, 127.52, 124.57, 124.31, 118.32 (Ph), 115.74 (C-4, C-12), 49.93 (C-3, C-13), 32.29 (C-5, C-11), 30.95 (C-8), 29.16 (CH_3_), 27.77 (CH_3_). Anal. calcd. for C_23_H_27_ClN_2_O_2_: C, 69.25; H, 6.82; N, 7.02%. Found; C, 69.15; H, 6.92; N, 7.03%. ESI-MS (*m/z*); M + 1 = 399.18, M + 2 = 400.17.
***10-amino-9-(4-bromophenyl)-3,3,6,6-tetramethyl-3,4,6,7-tetrahydroacridine-1,8(2H,5H,9H,10H)-dione (*4g*)*:** Yield 95%, pale yellow crystalline solid, m.p. 221–223 °C. IR Spectrum (υ, cm^−1^): 3350.77 (N-H), 3086.1 (C=C-H), 2979, 2959 (*sp^3^* C-H), and 1625.16 (C=O). ^1^H NMR spectrum (CDCl_3_, 500 MHz): 6.94–6.96 (d, 2H, H-3′, H-5′), 7.26–7.38 (d, 2H, H-2′, H-6′), 5.29 (s, 2H, NH_2_), 4.97 (s, 1H, H-8), 2.90–1.06 (*m*, 8H, 2*H-3, 2*H-5, 2*H-11, 2*H-13), 0.98 (s, 6H, 2*CH_3_), 0.86 (s, 6H, 2*CH_3_). ^13^C NMR spectrum (CDCl_3_, 125 MHz): 197.45 (C=O), 157.40 (C-2, C-14), 137.29 (C-7, C-9), 131.21, 128.57, 119.54 (Ph), 115.18 (C-4, C-12), 46.99 (C-3, C-13), 32.42 (C-5, C-11), 31.36 (C-8), 28.00 (CH_3_), 27.38 (CH_3_). Anal. calcd. for C_23_H_27_BrN_2_O_2_: C, 62.31; H, 6.14; N, 6.32%. Found; C, 62.51; H, 6.04; N, 6.44%. ESI-MS (*m/z*); M + 1 = 443.14, M + 2 = 444.13.
***10-amino-9-(4-florophenyl)-3,3,6,6-tetramethyl-3,4,6,7-tetrahydroacridine-1,8(2H,5H,9H,10H)-dione (*4h*)*:** Yield 93%, pale yellow crystalline solid, m.p. 297–299 °C. IR Spectrum (υ, cm^−1^): 3339.23 (N-H), 3065.1 (C=C-H), 2929, 2909 (*sp^3^* C-H), and 1616.16 (C=O). ^1^H NMR spectrum (CDCl_3_, 500 MHz): 7.14 (d, 2H, H-3′, H-5′), 7.01 (d, 2H, H-2′, H-6′), 5.29 (s, 2H, NH_2_), 4.94 (s, 1H, H-8), 2.90–2.01 (*m*, 8H, 2*H-3, 2*H-5, 2*H-11, 2*H-13), 1.02 (s, 6H, 2*CH_3_), 0.86 (s, 6H, 2*CH_3_). ^13^C NMR spectrum (CDCl_3_, 125 MHz): 194.5 (C=O), 154.23 (C-2, C-14), 145.2 (C-7, C-9), 129.90, 128.49, 119.20, 111.38 (Ph), 115.15 (C-4, C-12), 49.47 (C-3, C-13), 31.50 (C-5, C-11), 30.26 (C-8), 29.38 (CH_3_), 26.57 (CH_3_). Anal. calcd. for C_23_H_27_FN_2_O_2_: C, 72.23; H, 7.12; N, 7.32%. Found; C, 72.43; H, 7.25; N, 7.13%. ESI-MS (*m/z*); M + 1 = 383.21, M + 2 = 384.21.
***10-amino-9-(4-methoxyphenyl)-3,3,6,6-tetramethyl-3,4,6,7-tetrahydroacridine-1,8(2H,5H,9H,10H)-dione (*4i*)*:** Yield 91%, pale yellow crystalline solid, m.p. 245–246 °C. IR Spectrum (υ, cm^−1^): 3327.77 (N-H), 3100.1 (C=C-H), 2945, 2920 (*sp^3^* C-H), and 1591.16 (C=O). ^1^H NMR spectrum (CDCl_3_, 500 MHz): 7.24–7.20 (d, 4H, H-2′, H-3′, H-5′, H-6′), 5.39 (s, 2H, NH_2_), 4.81 (s, 1H, H-8), 2.91–1.98 (*m*, 8H, 2*H-3, 2*H-5, 2*H-11, 2*H-13), 1.10 (s, 6H, 2*CH_3_), 0.96 (s, 6H, 2*CH_3_). ^13^C NMR spectrum (CDCl_3_, 125 MHz): 195.34 (C=O), 153.40 (C-2, C-14), 147.44 (C-7, C-9), 126.20, 126.05, 125.78 (Ph), 115.55 (C-4, C-12), 48.40 (C-3, C-13), 39.25 (C-5, C-11), 33.62 (C-8), 29.78 (CH_3_), 26.69 (CH_3_). Anal. calcd. for C_24_H_30_N_2_O_3_: C, 73.07; H, 7.66; N, 7.10%. Found; C, 73.5; H, 7.60; N, 7.13%. ESI-MS (*m/z*); M + 1 = 395.23.
***10-amino-9-(3-methoxyphenyl)-3,3,6,6-tetramethyl-3,4,6,7-tetrahydroacridine-1,8(2H,5H,9H,10H)-dione (*4j*)*:** Yield 93%, pale yellow crystalline solid, m.p. 229–231 °C. IR Spectrum (υ, cm^−1^): 3320.67 (N-H), 3105.1 (C=C-H), 2949, 2935 (*sp^3^* C-H), and 1593.16 (C=O). ^1^H NMR spectrum (CDCl_3_, 500 MHz): 7.35–7.33 (d, 1H, H-4′), 7.17–7.13 (t, 1H, H-5′), 7.10–7.07 (d, 1H, H-6′), 7.05–7.020 (s, 1H, H-2′), 5.170 (s, 2H, NH_2_), 4.62 (s, 1H, H-8), 2.85–1.97 (*m*, 8H, 2*H-3, 2*H-5, 2*H-11, 2*H-13), 1.11 (s, 6H, 2*CH_3_), 0.97 (s, 6H, 2*CH_3_). ^13^C NMR spectrum (CDCl_3_, 125 MHz): 195.29 (C=O), 153.49 (C-2, C-14), 147.39 (C-7, C-9), 126.67, 126.25, 125.81 (Ph), 115.13 (C-4, C-12), 48.54 (C-3, C-13), 39.56 (C-5, C-11), 33.25 (C-8), 29.81 (CH_3_), 26.79 (CH_3_). Anal. calcd. for C_24_H_30_N_2_O_3_: C, 73.07; H, 7.66; N, 7.10%. Found; C, 73.5; H, 7.60; N, 7.13%. ESI-MS (*m/z*); M + 1 = 395.23.
***10-amino-9-(3,4-dimethoxyphenyl)-3,3,6,6-tetramethyl-3,4,6,7-tetrahydroacridine-1,8(2H,5H,9H,10H)-dione (*4k*)*:** Yield 89%, pale yellow crystalline solid, m.p. 211–213 °C. IR Spectrum (υ, cm^−1^): 3326.77 (N-H), 3046.1 (C=C-H), 2959, 2929 (*sp^3^* C-H), and 1615.16 (C=O). ^1^H NMR spectrum (CDCl_3_, 500 MHz): 7.14–7.13 (d, 4H, H-5′), 7.81–7.77 (s, 1H, H-2′), 7.05–7.0250 (d, 1H, H-6′), 5.28 (s, 2H, NH_2_), 4.95 (s, 1H, H-8), 2.91–1.99 (*m*, 8H, 2*H-3, 2*H-5, 2*H-11, 2*H-13), 1.02 (s, 6H, 2*CH_3_), 0.86 (s, 6H, 2*CH_3_). ^13^C NMR spectrum (CDCl_3_, 125 MHz): 194.44 (C=O), 154.47 (C-2, C-14), 146.33 (C-7, C-9), 127.49, 127.20, 125.38 (Ph), 115.27 (C-4, C-12), 49.47 (C-3, C-13), 38.50 (C-5, C-11), 31.26 (C-8), 29.38 (CH_3_), 26.57 (CH_3_). Anal. calcd. for C_25_H_32_N_2_O_4_: C, 70.73; H, 7.60; N, 6.60%. Found; C, 70.78; H, 7.56; N, 6.56%. ESI-MS (*m/z*); M + 1 = 425.24.
***10-amino-3,3,6,6-tetramethyl-9-p-tolyl-3,4,6,7-tetrahydroacridine-1,8(2H,5H,9H,10H)-dione (*4l*)*:** Yield 92%, pale yellow crystalline solid, m.p. 279–280 °C. IR Spectrum (υ, cm^−1^): 3316.77 (N-H), 3053.1 (C=C-H), 2945, 2927 (*sp^3^* C-H), and 1601.16 (C=O). ^1^H NMR spectrum (CDCl_3_, 500 MHz): 7.72–7.82 (d, 2H, H-3′, H-5′), 7.26–7.35 (d, 2H, H-2′, H-6′), 5.29 (s, 1H, H-8), 4.90 (s, 2H, NH_2_), 2.31–2.51 (*m*, 8H, 2*H-3, 2*H-5, 2*H-11, 2*H-13), 2.32 (s, 3H, C-4′-CH_3_), 1.23 (s, 6H, 2^*^CH_3_), 1.11 (s, 6H, 2^*^CH_3_) ppm. ^13^C NMR spectrum (CDCl_3_, 125 MHz): 197.51 (C=O), 160.47 (C-2, C-14), 138.10 (C-7, C-9), 128.22, 126.80, 125.85, 119.69 (Ph), 115.60 (C-4, C-12), 47.08 (C-3, C-13), 46.46 (C-5, C-11), 31.43 (C-8), 29.66 (CH_3_), 25.95 (CH_3_). Anal. calcd. for C_24_H_30_N_2_O_2_: C, 76.16; H, 7.99; N, 7.40%. Found; C, 76.10; H, 7.93; N, 7.44. ESI-MS (*m/z*); M + 1 = 379.23.
***10-amino-3,3,6,6-tetramethyl-9-m-tolyl-3,4,6,7-tetrahydroacridine-1,8(2H,5H,9H,10H)-dione (*4m*)*:** Yield 93%, pale yellow crystalline solid, m.p. 241–243 °C. IR Spectrum (υ, cm^−1^): 3310.65 (N-H), 3049.3 (C=C-H), 2950, 2931 (*sp^3^* C-H), and 1604.23 (C=O). ^1^H NMR spectrum (CDCl_3_, 500 MHz): 7.07 (d, 1H, H-4′), 7.02 (t, 1H, H-5′), 7.01 (d, 1H, H-6′), 6.95 (s, J = 7.7 Hz, 1H, H-2′), 5.25 (s, 1H, H-8), 5.20 (s, 2H, NH_2_), 2.29 (*m*, 8H, 2*H-3, 2*H-5, 2*H-11, 2*H-13), 2.24 (s, 3H, C-3′-CH_3_), 1.29 (s, 6H, 2*CH3), 1.24 (s, 6H, 2*CH_3_) ppm. ^13^C NMR spectrum (CDCl_3_, 125 MHz): 195.44 (C=O), 159.47 (C-2, C-14), 142.33 (C-7, C-9), 129.49, 129.20, 128.38 (Ph), 115.53 (C-4, C-12), 46.29 (C-3, C-13), 45.39 (C-5, C-11), 30.43 (C-8), 29.73 (CH_3_), 20.43 (CH_3_). Anal. calcd. for C_24_H_30_N_2_O_2_: C, 76.16; H, 7.99; N, 7.40%. Found; C, 76.10; H, 7.93; N, 7.44%. ESI-MS (*m/z*); M + 1 = 379.23.
***10-amino-3,3,6,6-tetramethyl-9-styryl-3,4,6,7-tetrahydroacridine-1,8(2H,5H,9H,10H)-dione* *(*4n*)*:** Yield 81%, pale yellow crystalline solid, m.p. 211–213 °C. IR Spectrum (υ, cm^−1^): 3325.65 (N-H), 3040.3 (C=C-H), 2966, 2943 (*sp^3^* C-H), and 1614.23 (C=O). ^1^H NMR spectrum (CDCl_3_, 500 MHz): 7.54 (t, 1H, H-4′), 7.12 (t, 2H, H-3′, H-5′), 7.07 (d, 1H, H-2′, H-6′), 7.03 (d, 1H, H-2′’), 6.70 (t, 1H, H-1′’), 5.75 (d, 1H, H-8), 5.21 (s, 2H, NH_2_), 2.29 (*m*, 8H, 2*H-3, 2*H-5, 2*H-11, 2*H-13), 1.29 (s, 6H, 2*CH3), 1.24 (s, 6H, 2*CH3) ppm. ^13^C NMR spectrum (CDCl_3_, 125 MHz): 200.44 (C=O), 162.47 (C-2, C-14), 145.33 (C-7, C-9), 130.49, 129.50, 129.38 (Ph), 113.67 (C-4, C-12), 46.29 (C-3, C-13), 46.39 (C-5, C-11), 31.43 (C-8), 20.73 (CH_3_), 20.20 (CH_3_). Anal. calcd. for C_25_H_30_N_2_O_2_: C, 76.89; H, 7.74; N, 7.17%. Found; C, 76.85; H, 7.70; N, 7.09%. ESI-MS (*m/z*); M + 1 = 391.23.
***10-amino-9-(furan-2-yl)-3,3,6,6-tetramethyl-3,4,6,7-tetrahydroacridine-1,8(2H,5H,9H,10H)-dione* *(*4o*)*:** Yield 93%, pale yellow crystalline solid, m.p. 265–266 °C. IR Spectrum (υ, cm^−1^): 3310.77 (N-H), 3025.1 (C=C-H), 2969, 2930 (*sp^3^* C-H), and 1610.26 (C=O). ^1^H NMR spectrum (CDCl_3_, 500 MHz): 7.09–7.26 (d, 1H, H-3′), 6.85–6.87 (m, 1H, H-4′), 6.62–6.63 (d, 1H, H-5′), 5.29 (s, 2H, NH_2_), 4.97 (s, 1H, H-8), 2.22–2.43 (*m*, 8H, 2*H-3, 2*H-5, 2*H-11, 2*H-13), 2.54 (s, 2H, H-11), 2.17–2.14 (s, 2H, H-3), 2.02–1.99 (s, 2H, H-13), 1.02 (s, 6H, 2*CH_3_), 0.86 (s, 6H, 2*CH_3_). ^13^C NMR spectrum (CDCl_3_, 125 MHz): 190.38 (C=O), 146.31 (C-2, C-14), 143.59 (C-7, C-9), 129.74, 119.51, 115.78, 114.08 (furyl–C), 109.82 (C-4, C-12), 55.66 (C-3, C-13), 47.09 (C-5, C-11), 46.39 (C-8), 29.84 (CH_3_), 27.10 (CH_3_). Anal. calcd. for C_21_H_20_N_2_O_3_: C, 71.16; H, 7.39; N, 7.90%. Found C, 71.19; H, 7.29; N, 7.75%. ESI-MS (*m/z*); M + 1 = 385.24.
***10-amino-3,3,6,6-tetramethyl-9-(thiophen-2-yl)-3,4,6,7-tetrahydroacridine-1,8(2H,5H,9H,10H)-dione (*4p*)*:** Yield 95%, pale yellow crystalline solid, m.p. 243–244 °C. IR Spectrum (υ, cm^−1^): 3317.77 (N-H), 3029.1 (C=C-H), 2975, 2830 (*sp^3^* C-H), and 1615.26 (C=O). ^1^H NMR spectrum (CDCl_3_, 500 MHz): 7.26 (d, 1H, H-3′), 6.79–6.80 (m, 1H, H-4′), 6.56–6.61 (d, 1H, H-5′), 5.31 (s, 2H, NH_2_), 4.98 (s, 1H, H-8), 2.29–2.43 (*m*, 8H, 2*H-3, 2*H-5, 2*H-11, 2*H-13), 1.23 (s, 6H, 2*CH_3_), 1.07 (s, 6H, 2*CH_3_). ^13^C NMR spectrum (CDCl_3_, 125 MHz): 189.86 (C=O), 143.67 (C-2, C-14), 146.36 (C-7, C-9), 126.31, 124.51, 123.44 (thiolphenyl–C), 109.87 (C-4, C-12), 46.98 (C-3, C-13), 31.13 (C-5, C-11), 30.37 (C-8), 29.91 (CH_3_), 26.73 (CH_3_). Anal. calcd. for C_21_H_26_N_2_O_2_S: C, 68.08; H, 7.07; N, 7.56%. Found C, 68.19; H, 7.11; N, 7.48%. ESI-MS (*m/z*); M + 1 = 371.17.
***10-amino-3,3,6,6-tetramethyl-9-(pyridin-2-yl)-3,4,6,7-tetrahydroacridine-1,8(2H,5H,9H,10H)-dione (*4q*)*:** Yield 89%, pale yellow crystalline solid, m.p. 278–280 °C. IR Spectrum (υ, cm^−1^): 3326.77 (N-H), 3046.1 (C=C-H), 2959, 2929 (*sp^3^* C-H), and 1615.16 (C=O). ^1^H NMR spectrum (CDCl_3_, 500 MHz): 7.27 (d, J=7.4 Hz, 1H, H-6′), 7.20 (dd, 1H, H-4′), 7.14 (dd, 1H, H-5′), 7.08 (d, J=7.0 Hz, 1H, H-3′), 5.28 (s, 2H, NH_2_), 4.95 (s, 1H, H-8), 2.91–1.99 (*m*, 8H, 2*H-3, 2*H-5, 2*H-11, 2*H-13), 1.02 (s, 6H, 2*CH_3_), 0.86 (s, 6H, 2*CH_3_). ^13^C NMR spectrum (CDCl_3_, 125 MHz): 194.44 (C=O), 154.47(C-2, C-14), 146.33 (C-7, C-9), 127.49, 127.20, 125.38 (Ph), 112.47 (C-4, C-12), 49.47 (C-3, C-13), 38.50 (C-5, C-11), 31.26 (C-8), 29.38 (CH_3_), 26.57 (CH_3_). Anal. calcd. for C_22_H_27_N_3_O_2_: C, 72.30; H, 7.45; N, 11.50%. Found; C, 72.35; H, 7.39; N, 11.46%. ESI-MS (*m/z*); M + 1 = 366.24.
***10-amino-3,3,6,6,9-pentamethyl-3,4,6,7-tetrahydroacridine-1,8(2H,5H,9H,10H)-dione* *(*4r*)*:** Yield 91%, pale yellow crystalline solid, m.p. 295–297 °C. IR Spectrum (υ, cm^−1^): 3315.77 (N-H), 3046.1 (C=C-H), 2953, 2930 (*sp^3^* C-H), and 1625.16 (C=O). ^1^H NMR spectrum (CDCl_3_, 500 MHz): 5.38 (s, 2H, NH_2_), 2.35 (*m*, 8H, 2*H-3, 2*H-5, 2*H-11, 2*H-13), 1.25 (s, 6H, 2*CH3), 1.21 (s, 6H, 2*CH3) ppm. ^13^C NMR spectrum (CDCl_3_, 125 MHz): 196.44 (C=O), 163.47 (C-2, C-14), 143.33 (C-7, C-9), 111.27 (C-4, C-12), 47.79 (C-3, C-13), 46.45 (C-5, C-11), 29.41 (CH_3_), 20.95 (CH_3_). Anal. calcd. for C_19_H_28_N_2_O_2_: C, 72.12; H, 8.93; N, 8.85%. Found; C, 72.16; H, 8.98; N, 8.83%. ESI-MS (*m/z*); M + 1 = 317.22.
***10-amino-3,3,6,6-tetramethyl-9-propyl-3,4,6,7-tetrahydroacridine-1,8(2H,5H,9H,10H)-dione* *(*4s*)*:** Yield 89%, pale yellow crystalline solid, m.p. 216–217 °C. IR Spectrum (υ, cm^−1^): 3315.77 (N-H), 3046.1 (C=C-H), 2953, 2930 (*sp^3^* C-H), and 1625.16 (C=O). ^1^H NMR spectrum (CDCl_3_, 500 MHz): 5.38 (*s*, 2H, NH_2_), 5.1 (*s*, 1H, H-4), 2.35 (*m*, 8H, H-3, H-5′, H-11, H-13), 1.25 (*s*, 6H, 2*CH_3_), 1.21 (*s*, 6H, 2*CH_3_) ppm, 1.01 (*m*, 2H, CH_2_), 0.92 (m, 2H, CH_2_), 0.73 (t, 3H, CH_3_). ^13^C NMR spectrum (CDCl_3_, 125 MHz): 196.44 (C=O), 163.47 (C-2, C-14), 143.33 (C-7, C-9), 113.34 (C-4, C-12), 47.79 (C-3, C-13), 46.45 (C-5, C-11), 29.41 (CH_3_), 20.95 (CH_3_). Anal. calcd. for C_21_H_32_N_2_O_2_: C, 73.22; H, 9.36; N, 8.13%. Found; C, 73.26; H, 9.41; N, 8.17%. ESI-MS (*m/z*); M + 1 = 345.25.

### 4.2. General Experimental Procedure for Preparation of 10-Amino-3,3,6,6-Tetramethyl-9-Phenyl-3,4,6,7-Tetrahydroacridine-1,8(2H,5H,9H,10H)-Dione (***4a***)

A mixture of 5,5-dimethylcyclohexane-1,3-dione **1** (10 mmol), benzaldehyde **2a** (5 mmol), and hydrazine hydrate **3** (5 mmol) was added in a round bottom flask containing *β*-CD (5 mmol%) as a catalyst in ACN (5 mL). This mixture was stirred at 80 °C for constantly 5–6 h. The reaction was constantly observed by TLC (*n*-hexane: ethyl acetate/4:6). After the completion of the reaction, it was cooled to room temperature, followed by filtration of the obtained solid. The solid was then recrystallized with ethanol to obtain a pure compound **4a** in 96% yield.

### 4.3. Antimicrobial Activity

The recently synthesized N-PHQ derivatives, namely **4a**–**s**, were subjected to testing against three Gram-negative bacteria strains (*Staphylococcus aureus* MTCC 96, *Klebsiella pneumonia* MTCC 3384, and *Escherichia coli* MTCC 443), two Gram-positive bacteria strains (*Streptococcus pyogenes* MTCC 442 and *Bacillus subtilis* MTCC 441), and three fungal strains (*Aspergillus Niger* MTCC 281, *Aspergillus Janus* MTCC 2751, and *Aspergillus sclerotium* MTCC 1008). To prepare the bacterial samples, they were cultured at 37 °C for 24 h and then stored in nutrient broth. Before inoculation, at 28 °C for 72 h, the fungal strains were grown in malt extract. A sequential dilution protocol was employed to test triplicates of each synthesized chemical at concentrations of 2, 4, 8, 16, 32, 64, and 128 g/mL the chemicals were dissolved in DMSO.

## 5. Conclusions

The authors have described an effective method for synthesizing various N-PHQs through Hantzsch condensation using *β*-CD as an efficient catalyst. This process demonstrates flexibility and diversity while achieving high yields (up to 96%) using readily available starting materials. The synthesized N-PHQs derivatives (19 compounds) were tested against six bacterial and four fungal strains using the serial dilution method as compared to amoxicillin and fluconazole as standard drugs. All the synthesized N-PHQs showed good resistance against the tested strains. Interestingly, the presence of polar groups (-NO_2_ in compounds **4b** and **4c** and -OMe in compounds **4i**, **4j**, and **4k**) on the phenyl ring, which can form hydrogen bonds with the microbe’s protein, displayed excellent antimicrobial activity, which is comparable to the standard drugs at MIC 4 μg/mL. It has also been noted that replacing the phenyl group with an alkyl group (as in compound **4r** and at MIC 4 μg/mL) significantly reduces the effectiveness of the compound against these strains, with MIC values increasing to 64 and 128 μg/mL.

## Figures and Tables

**Table 1:** Effect of Different Catalyst–Solvent Combinations on the Synthesis of Compound **4a**.

Catalyst ^a^	Solvent	Time (hours)	Yield ^b^ (%)
-	Ethanol	9	52
-	ACN	10	45
-	Diethyl ether	8	23
Piperidine	Ethanol	12	15
Piperidine	ACN	11	40
Piperidine	Diethyl ether	8.5	42
SDS	H_2_O	11.5	36
Al(DS)_3_	H_2_O	13	47
Sc(DS)_3_	H_2_O	13	43
*β*-CD	Ethanol	6	74
*β*-CD	ACN	6	96
*β*-CD	Diethyl ether	10	65
2-aminopyrazine	Ethanol	8	No product
2-aminopyrazine	ACN	12	No product
2-aminopyrazine	Diethyl ether	10	No product

^a^ Amount of catalyst is 5 mmol%; the solvent is 5 mL. ^b^ Yield refers to the cumulative production output of all crops. Reaction conditions: 5,5-dimethylcyclohexane-1,3-dione **1** (10 mmol), benzaldehyde **2a** (5 mmol), and hydrazine hydrate **3** (5 mmol) refluxed at 80 °C.

**Table 2:** Standardization of Temperature for the Synthesis of Compound **4a** Using *β*-CD (5 mmol%) in ACN (5 mL).

Temperature (°C)	Time (hours)	Yield * (%)
50	>10	45
55	10	45
60	7	48
65	7	50
70	7	52
75	6.5	76
80	6	96
85	6	94
90	5	90
95	5	90

* Yield refers to the cumulative production output of all crops.

**Table 3:** Synthesis of N-PHQ Derivatives **4a**–**s** Using *β*-CD (5 mmol%) in ACN (5 mL).

Product	R	R_f_ ^a^	Melting Point (°C)		Yield ^b^ (%)
			Literature	Observed	
**4a**	C_6_H_5_	0.68	260–262	260–261	96 [41]
**4b**	4-NO_2_ C_6_H_4_	0.79	270–272	270–272	95 [42]
**4c**	3-NO_2_ C_6_H_4_	0.71	224–226	223–225	92 [41]
**4d**	2-NO_2_ C_6_H_4_	0.69	209–211	210–212	89 [41]
**4e**	4-Cl C_6_H_4_	0.55	274–276	274–275	94 [41]
**4f**	2-Cl C_6_H_4_	0.58	204–206	203–204	91 [41]
**4g**	4-Br C_6_H_4_	0.57	222–223	221–223	95 [42]
**4h**	4-F C_6_H_4_	0.61	>300	297–299	93 [42]
**4i**	4-OCH_3_ C_6_H_4_	0.59	244–245	245–246	91 [41]
**4j**	3-OCH_3_ C_6_H_4_	0.63	228–230	229–231	93 [41]
**4k**	3,4-(OCH_3_)_2_ C_6_H_4_	0.68	212–214	211–213	89 [41]
**4l**	4-CH_3_ C_6_H_4_	0.56	279–281	279–280	92 [41]
**4m**	3-CH_3_ C_6_H_4_	0.60	240–242	241–243	93 [41]
**4n**	C_6_H_5_CH=CH	0.57	213–215	211–213	81 [42]
**4o**	2-Furyl	0.64	264–265	265–266	93 [42]
**4p**	2-Thiophenyl	0.61	243–245	243–244	95 [42]
**4q**	2-Pyridyl	0.58	277–280	278–280	89 [41]
**4r**	CH_3_	0.63	296–297	295–297	91 [41]
**4s**	CH_3_CH_2_CH_2_	0.56	216–217	216–217	89 [42]

^a^ TLC solvent: *n*-hexane: ethyl acetate/4:6. ^b^ Yield refers to the cumulative production output of all crops.

**Table 4:** Minimum Inhibitory Concentration (μg/mL) of Synthesized N-PHQ Derivatives **4a**–**s** Against Various Microbial Agents.

Product	R	Gram (+ve) Bacteria		Gram (−ve) Bacteria			Fungi		
		*B. subtilis*	*S. pyogenes*	*E. coli*	*K. pneumonia*	*S. aureus*	*A. janus*	*A. niger*	*A. sclerotiorum*
**4a**	C_6_H_5_	16	8	8	8	16	8	16	8
**4b**	4-NO_2_ C_6_H_4_	8	4	8	4	8	16	8	16
**4c**	3-NO_2_ C_6_H_4_	4	4	4	8	4	4	4	8
**4d**	2-NO_2_ C_6_H_4_	8	8	16	8	16	16	16	8
**4e**	4-Cl C_6_H_4_	16	16	8	8	16	8	16	–
**4f**	2-Cl C_6_H_4_	64	16	32	16	32	32	32	16
**4g**	4-Br C_6_H_4_	16	32	32	32	16	32	32	32
**4h**	4-F C_6_H_4_	32	16	8	–	16	32	16	16
**4i**	4-OCH_3_ C_6_H_4_	8	4	8	4	8	16	8	16
**4j**	3-OCH_3_ C_6_H_4_	4	4	4	8	4	4	4	8
**4k**	3,4-(OCH_3_)_2_ C_6_H_4_	4	4	4	4	4	4	4	4
**4l**	4-CH_3_ C_6_H_4_	16	16	8	8	16	8	16	–
**4m**	3-CH_3_ C_6_H_4_	64	16	32	16	32	32	32	16
**4n**	C_6_H_5_CH=CH	16	32	32	32	16	32	32	32
**4o**	2-Furyl	32	16	8	–	16	32	16	16
**4p**	2-Thiophenyl	8	16	16	32	–	16	16	32
**4q**	2-Pyridyl	8	8	8	16	8	16	32	16
**4r**	CH_3_	64	64	64	32	128	64	128	64
**4s**	CH_3_CH_2_CH_2_	64	128	128	64	32	-	64	128
Amoxicillin Fluconazole		4	4	4	4	4	–	–	–
		–	–	–	–	–	2	2	2

**Table 5:** Comparison with Previous Literature Reports for the Synthesis of **4a**.

Entry	Catalyst	Conditions	Time (h)	Yield (%)
1	Silica iodide	EtOH/120 °C	2.5	90 [48]
2	KH_2_PO_4_	EtOH:H_2_O(3:1)/120 °C	5	94 [49]
3	FSG-Hf(NPf_2_)_4_	EtOH:H_2_O(1:1)/120 °C	4	82 [50]
4	*β*-CD	ACN/80 °C	6	96 (This work)

## Data Availability

Data are contained within the article and Supplementary Materials.

## Supplementary Materials

The following supporting information can be downloaded at: https://www.mdpi.com/article/10.3390/molecules29194655/s1, Figure S1. IR spectrum of *10-amino-3,3,6,6-tetramethyl-9-phenyl-3,4,6,7,9,10-hexahydroacridine-1,8(2H,5H)-dione (***4a***)*, Figure S2. ^1^H NMR spectrum of *10-amino-3,3,6,6-tetramethyl-9-phenyl-3,4,6,7,9,10-hexahydroacridine-1,8(2H,5H)-dione (***4a***)*, Figure S3. ^13^C NMR spectrum of *10-amino-3,3,6,6-tetramethyl-9-phenyl-3,4,6,7,9,10-hexahydroacridine-1,8(2H,5H)-dione (***4a***)*, Figure S4. DEPT-135 spectrum of *10-amino-3,3,6,6-tetramethyl-9-phenyl-3,4,6,7,9,10-hexahydroacridine-1,8(2H,5H)-dione (***4a***)*, Figure S5. DEPT-135 expanded spectrum of *10-amino-3,3,6,6-tetramethyl-9-phenyl-3,4,6,7,9,10-hexahydroacridine-1,8(2H,5H)-dione (***4a***)*, Figure S6. DEPT-135 expanded spectrum of *10-amino-3,3,6,6-tetramethyl-9-phenyl-3,4,6,7,9,10-hexahydroacridine-1,8(2H,5H)-dione (***4a***)*, Figure S7. HSQC spectrum of *10-amino-3,3,6,6-tetramethyl-9-phenyl-3,4,6,7,9,10-hexahydroacridine-1,8(2H,5H)-dione (***4a***)*, Figure S8. HSQC expanded spectrum of *10-amino-3,3,6,6-tetramethyl-9-phenyl-3,4,6,7,9,10-hexahydroacridine-1,8(2H,5H)-dione (***4a***)*, Figure S9. HSQC expanded spectrum of *10-amino-3,3,6,6-tetramethyl-9-phenyl-3,4,6,7,9,10-hexahydroacridine-1,8(2H,5H)-dione (***4a***)*, Figure S10. HSQC expanded spectrum of *10-amino-3,3,6,6-tetramethyl-9-phenyl-3,4,6,7,9,10-hexahydroacridine-1,8(2H,5H)-dione (***4a***)*, Figure S11. COSY expanded spectrum of *10-amino-3,3,6,6-tetramethyl-9-phenyl-3,4,6,7,9,10-hexahydroacridine-1,8(2H,5H)-dione (***4a***)*, Figure S12. COSY expanded spectrum of *10-amino-3,3,6,6-tetramethyl-9-phenyl-3,4,6,7,9,10-hexahydroacridine-1,8(2H,5H)-dione (***4a***)*, Figure S13. COSY expanded spectrum of *10-amino-3,3,6,6-tetramethyl-9-phenyl-3,4,6,7,9,10-hexahydroacridine-1,8(2H,5H)-dione (***4a***)*, Figure S14. COSY expanded spectrum of *10-amino-3,3,6,6-tetramethyl-9-phenyl-3,4,6,7,9,10-hexahydroacridine-1,8(2H,5H)-dione (***4a***)*, Figure S15. ^1^H NMR spectrum of *10-amino-3,3,6,6-tetramethyl-9-(4-nitrophenyl)-3,4,6,7-tetrahydroacridine-1,8(2H,5 H,9H,10H)- dione (***4b***)*, Figure S16. ^1^H NMR spectrum of *10-amino-3,3,6,6-tetramethyl-9-(3-nitrophenyl)-3,4,6,7-tetrahydroacridine-1,8(2H,5H,9H,10H)-dione (***4c***)*, Figure S17. ^1^H NMR spectrum of *10-amino-9-(2-chlorophenyl)-3,3,6,6-tetramethyl-3,4,6,7-tetrahydroacridine-1,8(2H*, *5H,9H,10H)-dione (***4f***)*, Figure S18. ^1^H NMR spectrum of *10-amino-9-(4-bromophenyl)-3,3,6,6-tetramethyl-3,4,6,7-tetrahydroacridine-1,8(2H*,*5H,9H,10H)-dione (***4g***)*, Figure S19. ^1^H NMR spectrum of *10-amino-9-(4-florophenyl)-3,3,6,6-tetramethyl-3,4,6,7-tetrahydroacridine-1,8(2H*, *5H,9H,10H)-dione (***4h***)*, Figure S20. ^1^H NMR spectrum of *10-amino-3,3,6,6-tetramethyl-9-p-tolyl-3,4,6,7-tetrahydroacridine-1,8(2H,5H,9H,10 H)-dione (***4l***)*, Figure S21. ^1^H NMR spectrum of *10-amino-9-(furan-2-yl)-3,3,6,6-tetramethyl-3,4,6,7-tetrahydroacridine-1,8(2H,5H*,*9H,10H)-dione (***4o***)*, Figure S22. ^1^H NMR spectrum of *10-amino-3,3,6,6-tetramethyl-9-(thiophen-2-yl)-3,4,6,7-tetrahydroacridine-1,8(2H,5 H,9H,10H)-dione (***4p***)*, Table S1. Impact of the amount of catalyst on the model reaction.
