# Supplementary material for: β-Cyclodextrin Catalyzed, One-Pot Multicomponent Synthesis and Antimicrobial Potential of N-Aminopolyhydroquinoline Derivatives
Source: Molecules. 2024 Sep 30;29(19):4655. doi: 10.3390/molecules29194655 (PMC11477876; doi:10.3390/molecules29194655)
Supplement: Supplementary file 1 [file molecules-29-04655-s001.zip › molecules-3177077-supplementary.pdf]

β-Cyclodextrin Catalyzed, One-pot Multicomponent Synthesis and Antimicrobial Potential of N-Aminopolyhydroquinoline Derivatives

Sonali Garg [1], Manvinder Kaur [1], Pradip K. Bhowmik [2], Harvinder Singh Sohal [1,*], Fohad Mabood Husain [3] and Haesook Han [2]

[1] Medicinal and Natural Product Laboratory, Department of Chemistry, Chandigarh University, Gharuan, Mohali 140413, Punjab, India
[2] Department of Chemistry and Biochemistry, University of Nevada Las Vegas, 4505 S. Maryland Parkway, Box 454003, Las Vegas, NV 89154, USA.
[3] Department of Food Science and Nutrition, College of Food and Agriculture Sciences, King Saud University, Riyadh 11451, Saudi Arabia
* Correspondence: drharvinder.cu@gmail.com

1. Results and discussion

1.1. Screening of the catalyst for the synthesis of N-Aminopolyhydroquinoline Derivatives

To optimize the conditions, we established the model reaction involves the condensation of 5,5-dimethylcyclohexane-1,3-dione **1** (2 eq.), benzaldehyde **2a** (1 eq.), and hydrazine hydrate **3** (1 eq.) refluxed at 80 ºC in the presence of β-CD catalyst for 6 hours in ethanol (5 mL) system to yield *10-amino-3,3,6,6-tetramethyl-9-phenyl-3,4,6,7-tetrahydroacridine-1,8(2H,5H,9H,10H)-dione* **4a**. We confirmed the reaction progress using TLC (*n*-hexane: ethyl acetate/4:6) and validated the product by comparing its melting point to literature data.

Initially, the influence of catalyst loading at different levels of catalytic loading was tested and observed that increasing the amount of catalyst increased the yield as shown in **Table 1**. The optimal amount of β-CD catalyst was found 5 mmol% **(Table 1, entry 3)**. Furthermore, the increase in the amount of β-CD catalyst exhibits minimal impact on percentage yield.

Table S1. Impact of the amount of catalyst on the model reaction.

Entry	β-CD (mmol%)	Yield* (%)
1	1	71%
2	3	83%
3	5	96%
4	7	92%
5	9	93%

*Yield refers to the cumulative production output of all crops.

2. Materials and Methods

All the chemicals used in this work were purchased from Sigma Aldrich and were used as such without further purification, while solvents were ordered from Loba Chemie. Melting points of all the products formed were taken on digital melting point apparatus *via* open capillary method. IR spectra of the targeted compound were taken using ATR mode on Perkin Elmer Spectrum II. ^{1}H-NMR and ^{13}C-NMR data of the synthesized compounds were recorded on a Bruker Advanced NEO 500 MHz NMR spectrometer, Saif, PU, Chandigarh, using solvent $CDCl_3$, and DMSO and using TMS as internal standard. Coupling constants are always expressed in unit Hertz [Hz]. 2D NMR (COSY and HSQC) were recorded on a Bruker Advanced NEO 500 MHz NMR spectrometer, Saif, PU, Chandigarh, using solvent $CDCl_3$, DMSO, and TMS as an internal standard. Coupling constants are always expressed in unit Hertz [Hz]. The thin layer chromatographic (TLC) technique was used during the reaction time to monitor the completion of the reaction and check the compound purity. A UV chamber was used for the visualization.

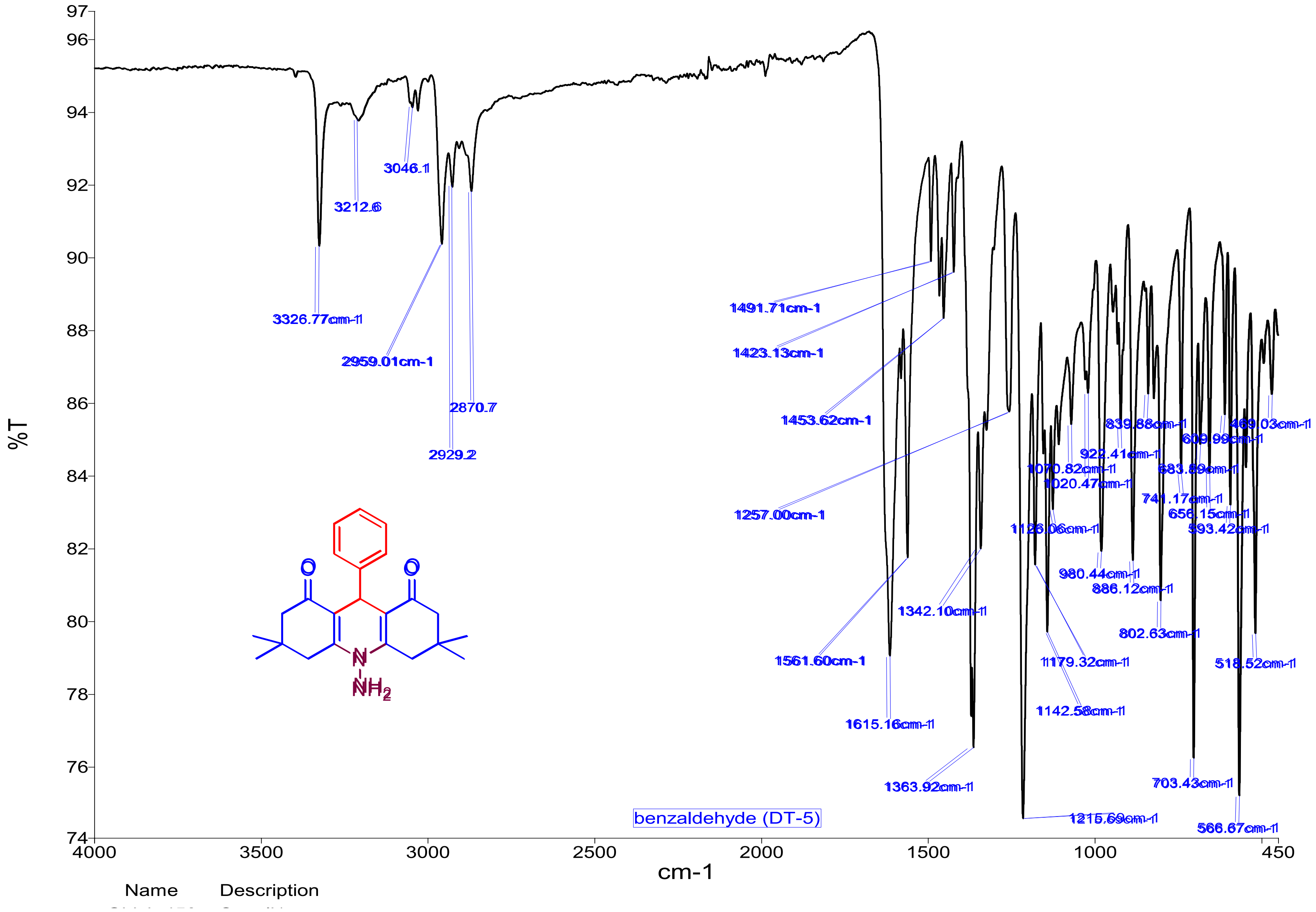

Figure S1. IR spectrum of *10-amino-3,3,6,6-tetramethyl-9-phenyl-3,4,6,7,9,10-hexahydroacridine-1,8(2H,5H)-dione* ***(4a)***.

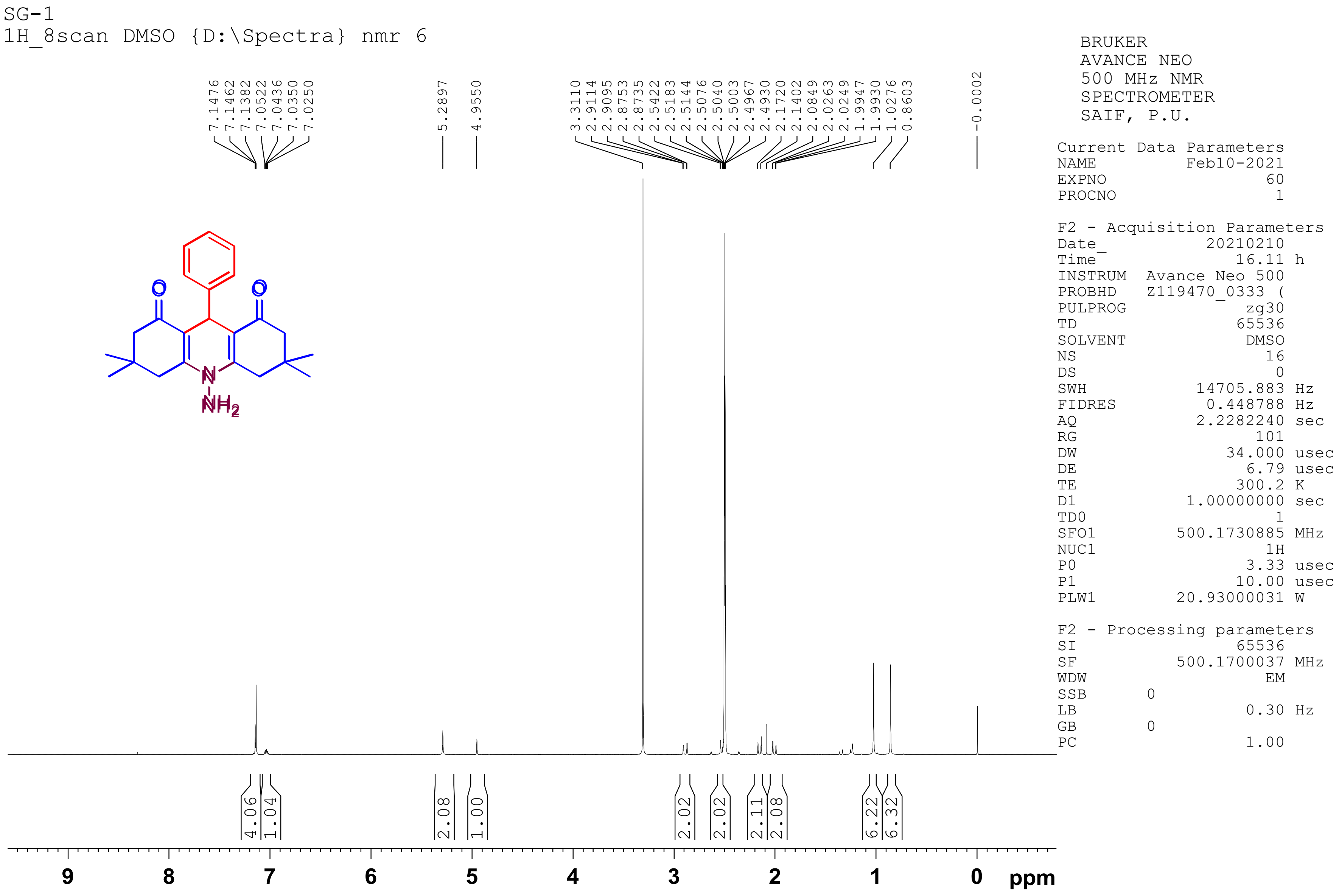

Figure S2. ^{1}**H NMR** spectrum of *10-amino-3,3,6,6-tetramethyl-9-phenyl-3,4,6,7,9,10-hexahydroacridine-1,8(2H,5H)-dione* ***(4a)***.

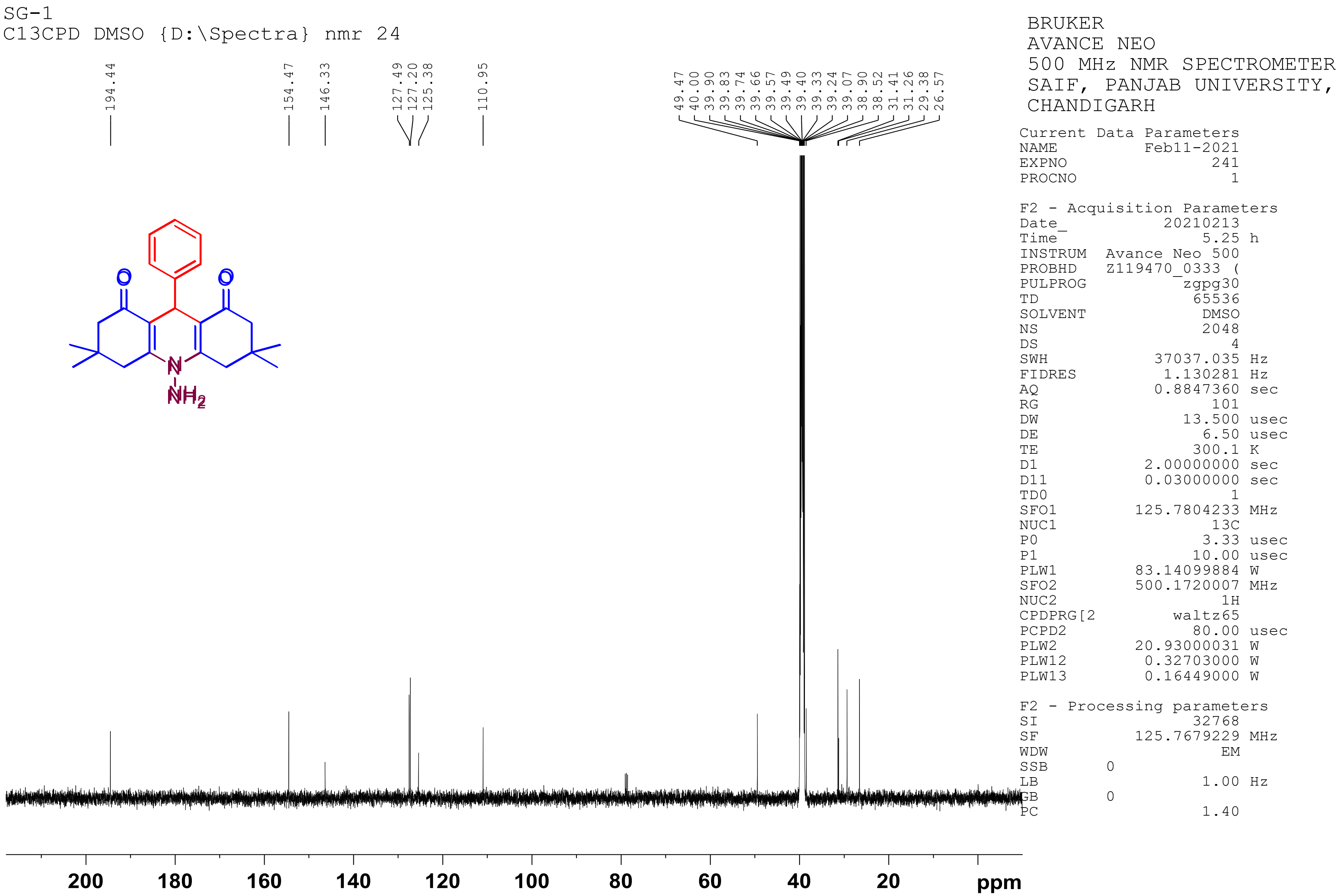

Figure S3. ^{13}**C NMR** spectrum of *10-amino-3,3,6,6-tetramethyl-9-phenyl-3,4,6,7,9,10-hexahydroacridine-1,8(2H,5H)-dione* ***(4a)***.

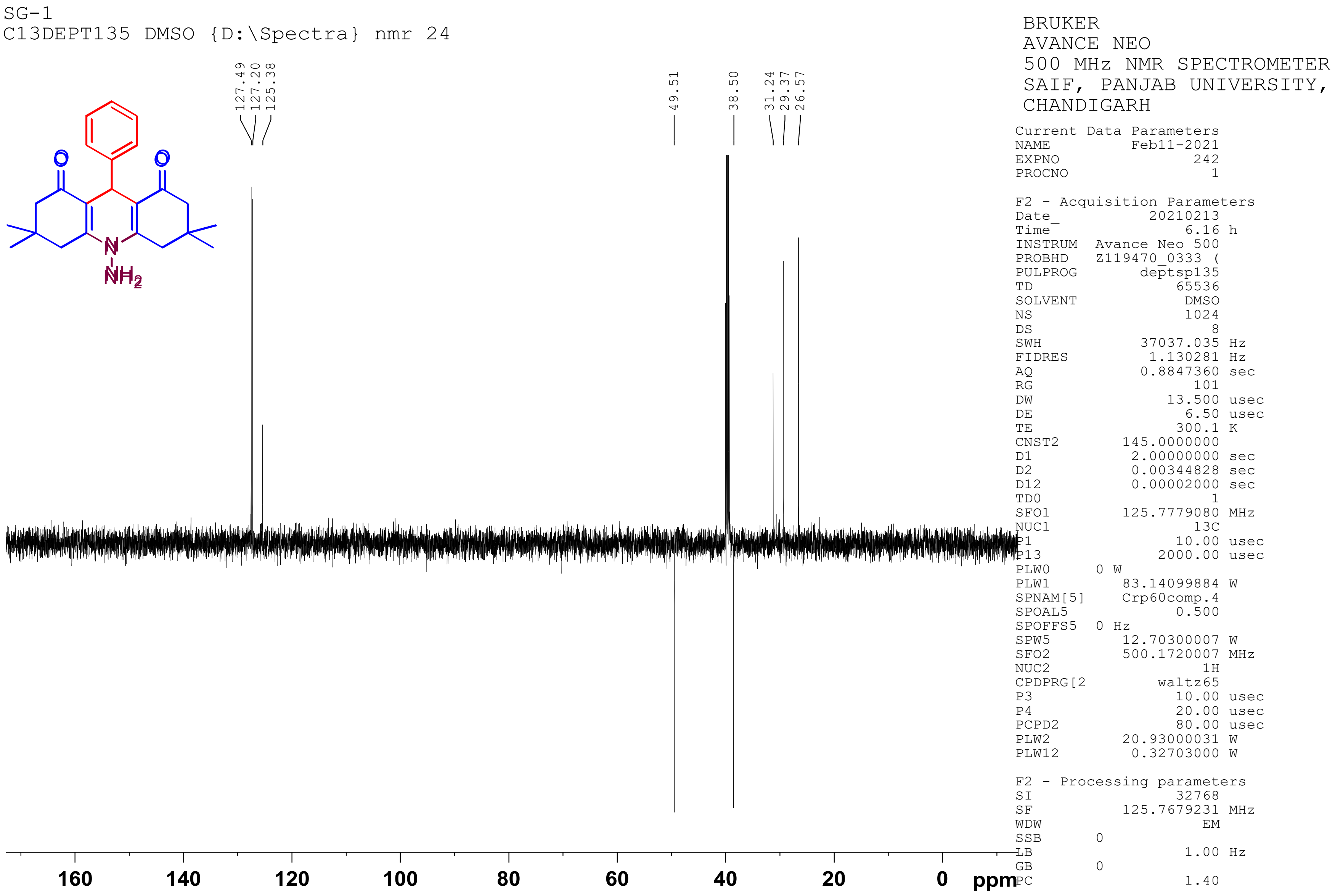

Figure S4. DEPT-135 spectrum of *10-amino-3,3,6,6-tetramethyl-9-phenyl-3,4,6,7,9,10-hexahydroacridine-1,8(2H,5H)-dione* ***(4a)***.

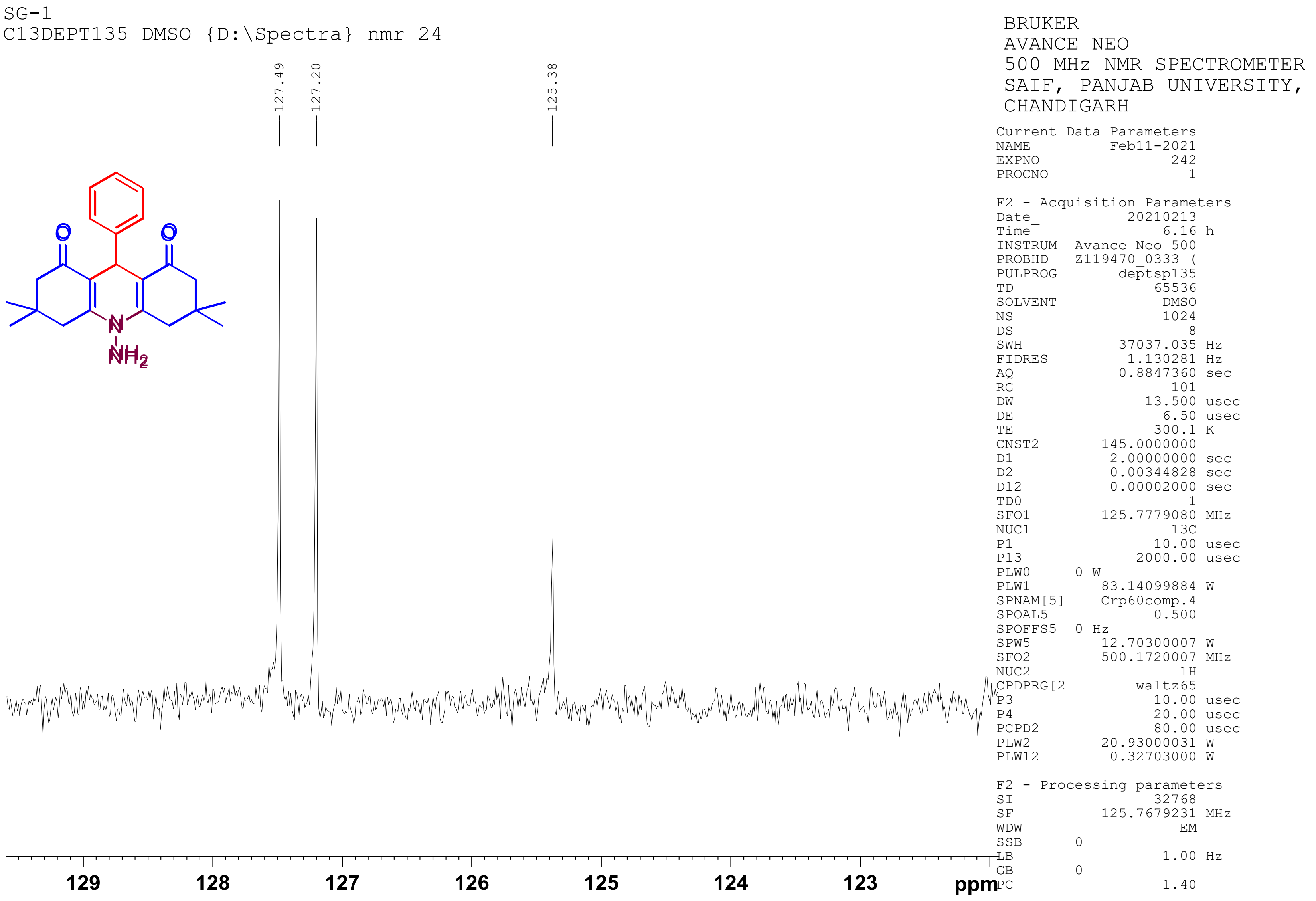

Figure S5. DEPT-135 expanded spectrum of *10-amino-3,3,6,6-tetramethyl-9-phenyl-3,4,6,7,9,10-hexahydroacridine-1,8(2H,5H)-dione* ***(4a)***.

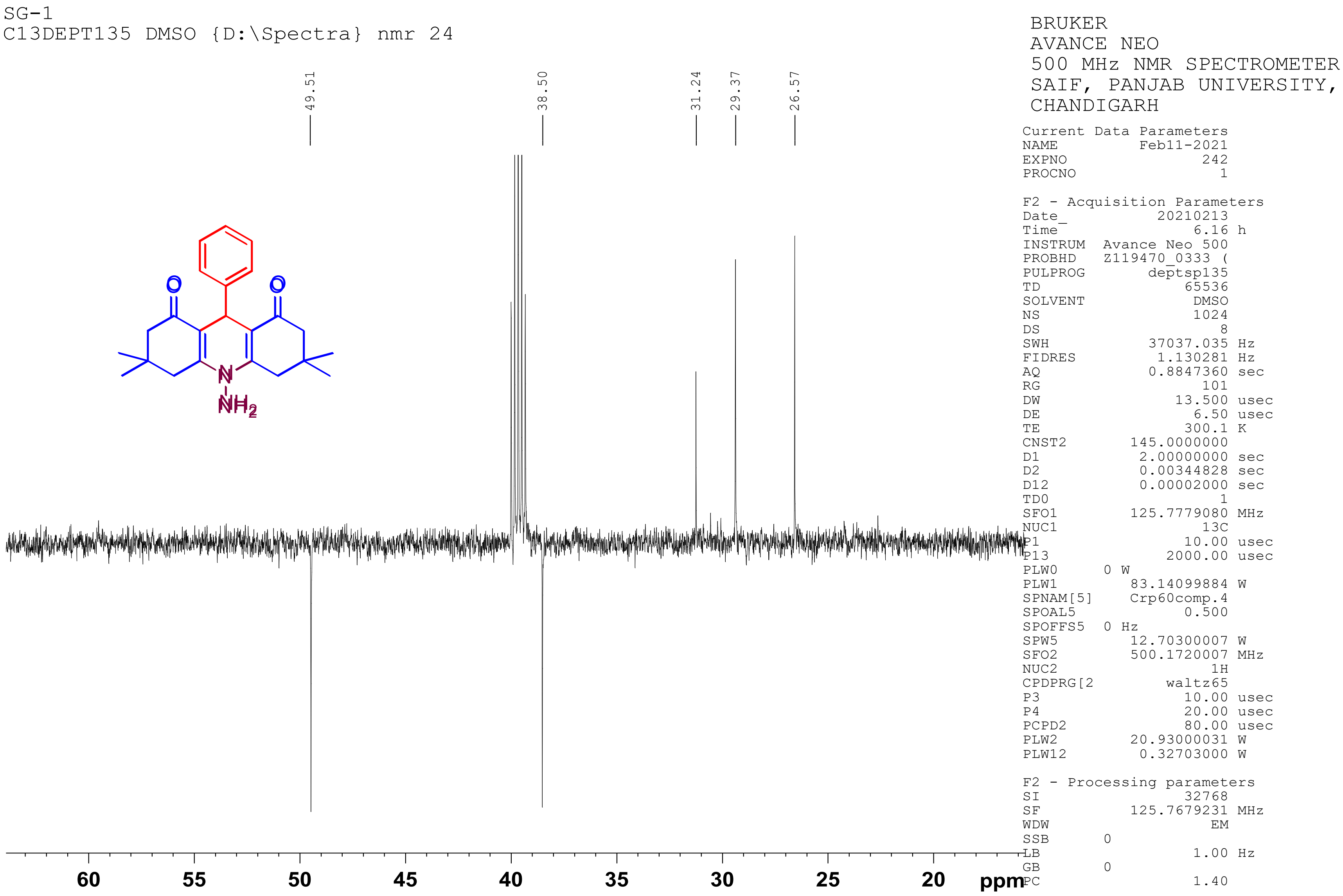

Figure S6. DEPT-135 expanded spectrum of *10-amino-3,3,6,6-tetramethyl-9-phenyl-3,4,6,7,9,10-hexahydroacridine-1,8(2H,5H)-dione* ***(4a)***.

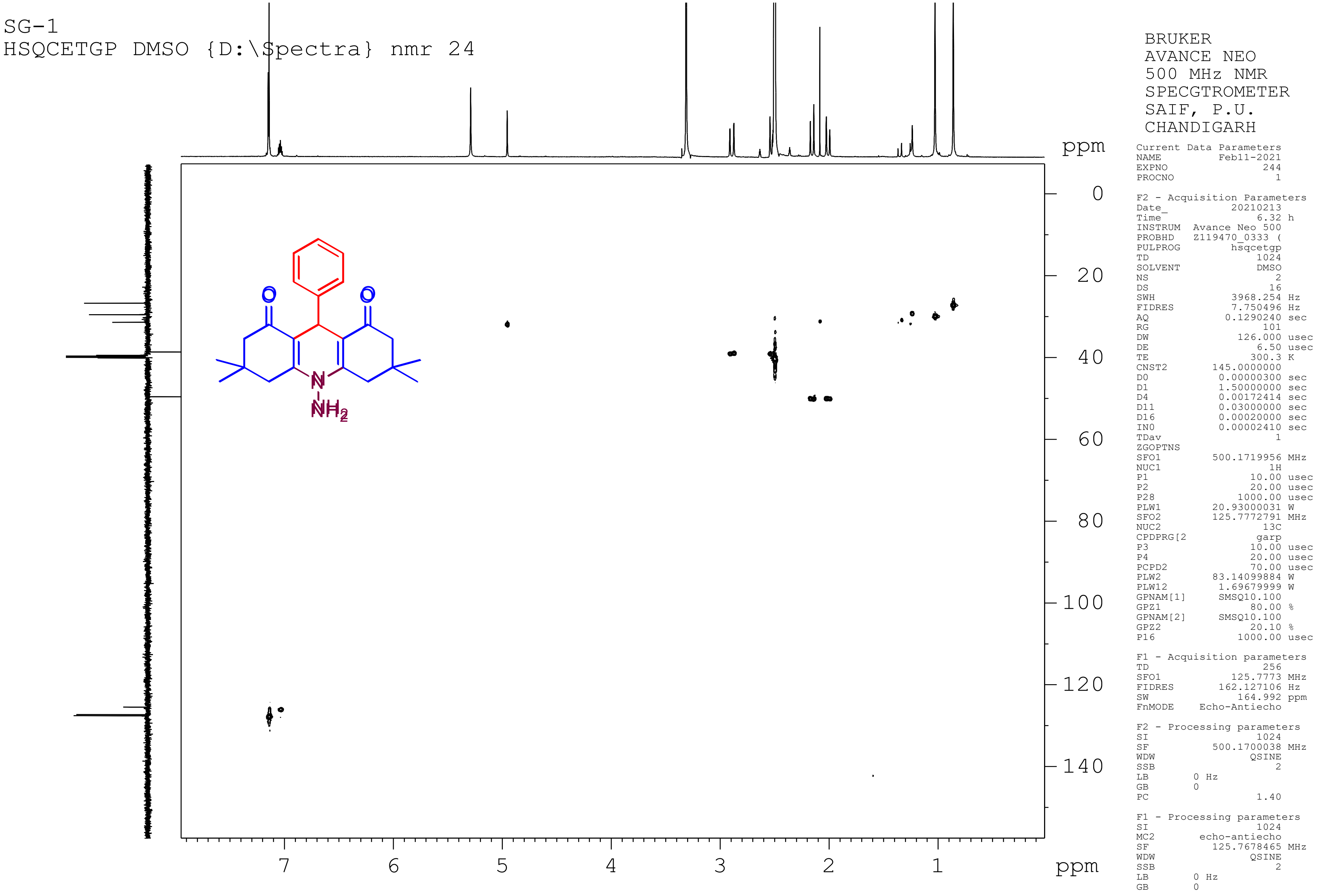

Figure S7. HSQC spectrum of *10-amino-3,3,6,6-tetramethyl-9-phenyl-3,4,6,7,9,10-hexahydroacridine-1,8(2H,5H)-dione* ***(4a)***.

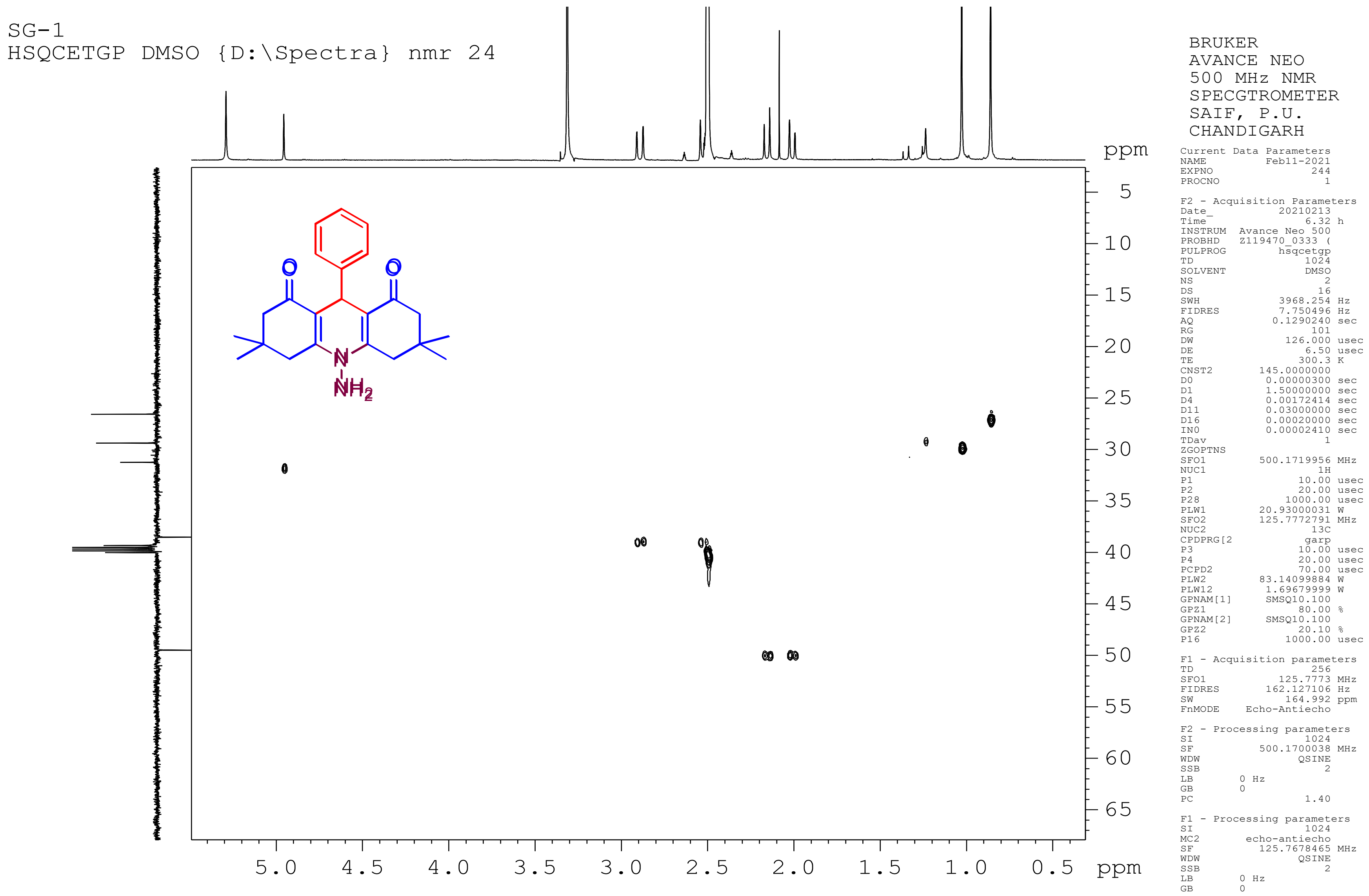

Figure S8. HSQC expanded spectrum of *10-amino-3,3,6,6-tetramethyl-9-phenyl-3,4,6,7,9,10-hexahydroacridine-1,8(2H,5H)-dione* ***(4a)***.

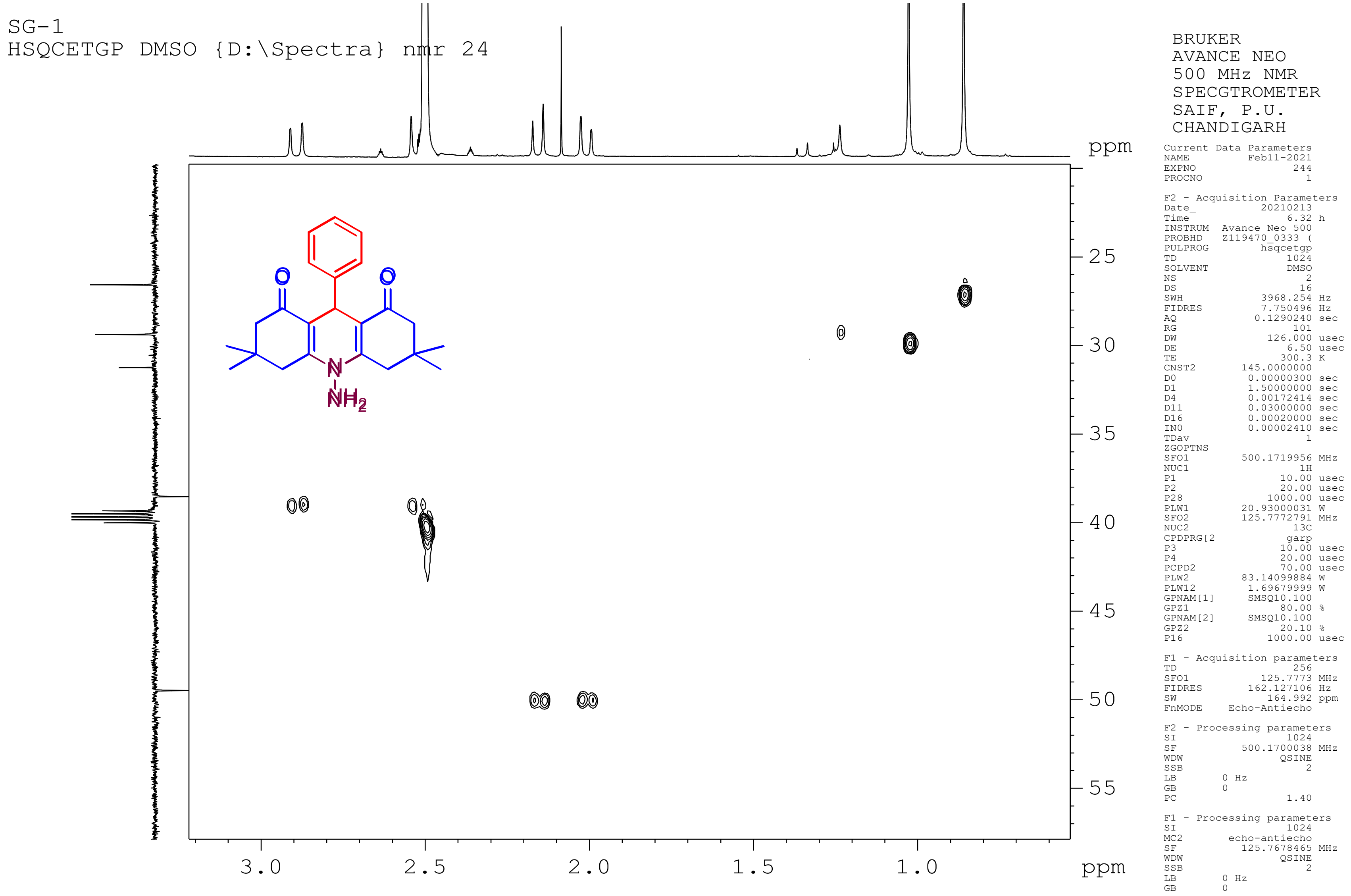

Figure S9. HSQC expanded spectrum of *10-amino-3,3,6,6-tetramethyl-9-phenyl-3,4,6,7,9,10-hexahydroacridine-1,8(2H,5H)-dione (4a)*.

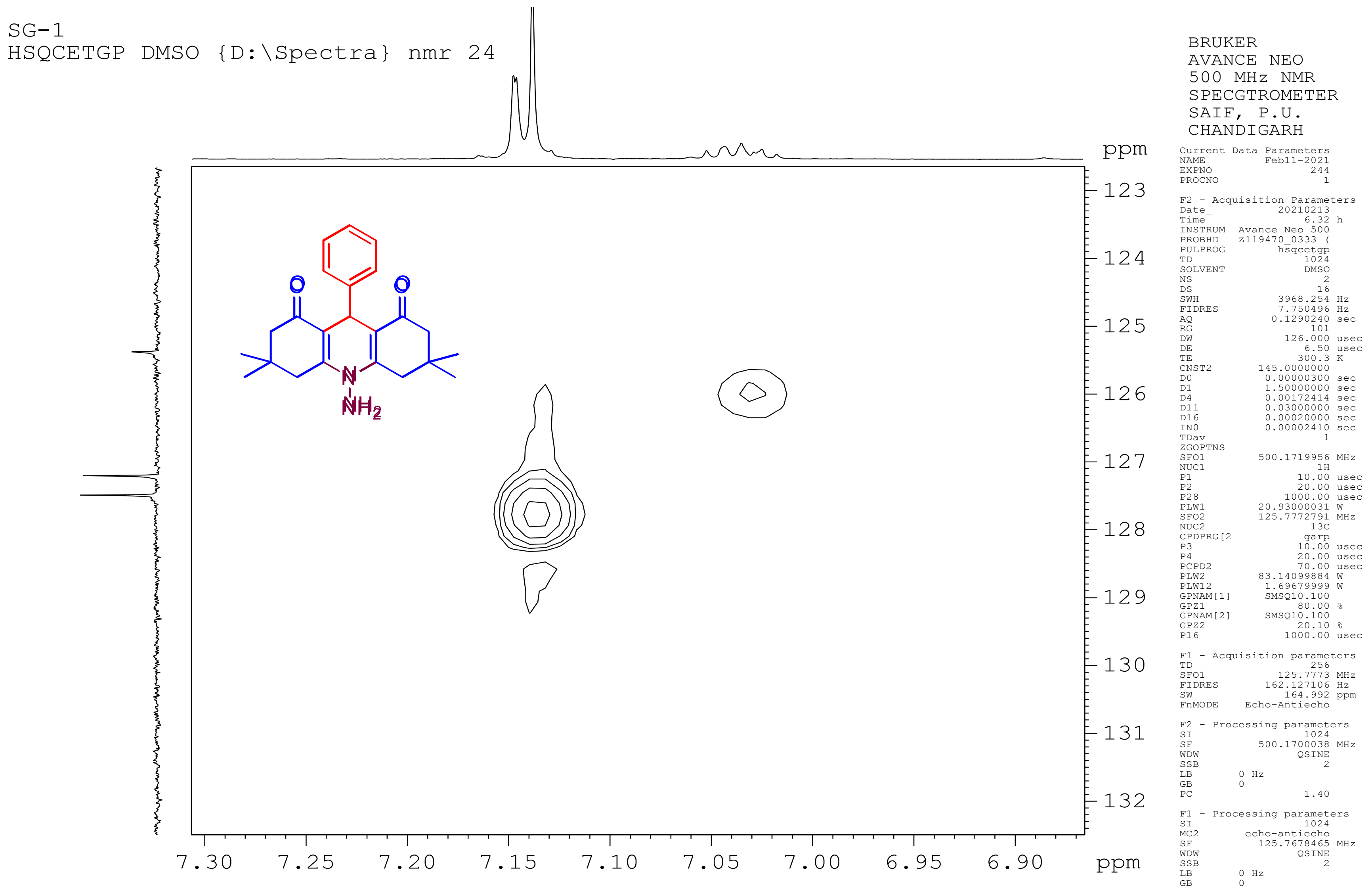

Figure S10. HSQC expanded spectrum of *10-amino-3,3,6,6-tetramethyl-9-phenyl-3,4,6,7,9,10-hexahydroacridine-1,8(2H,5H)-dione (4a)*.

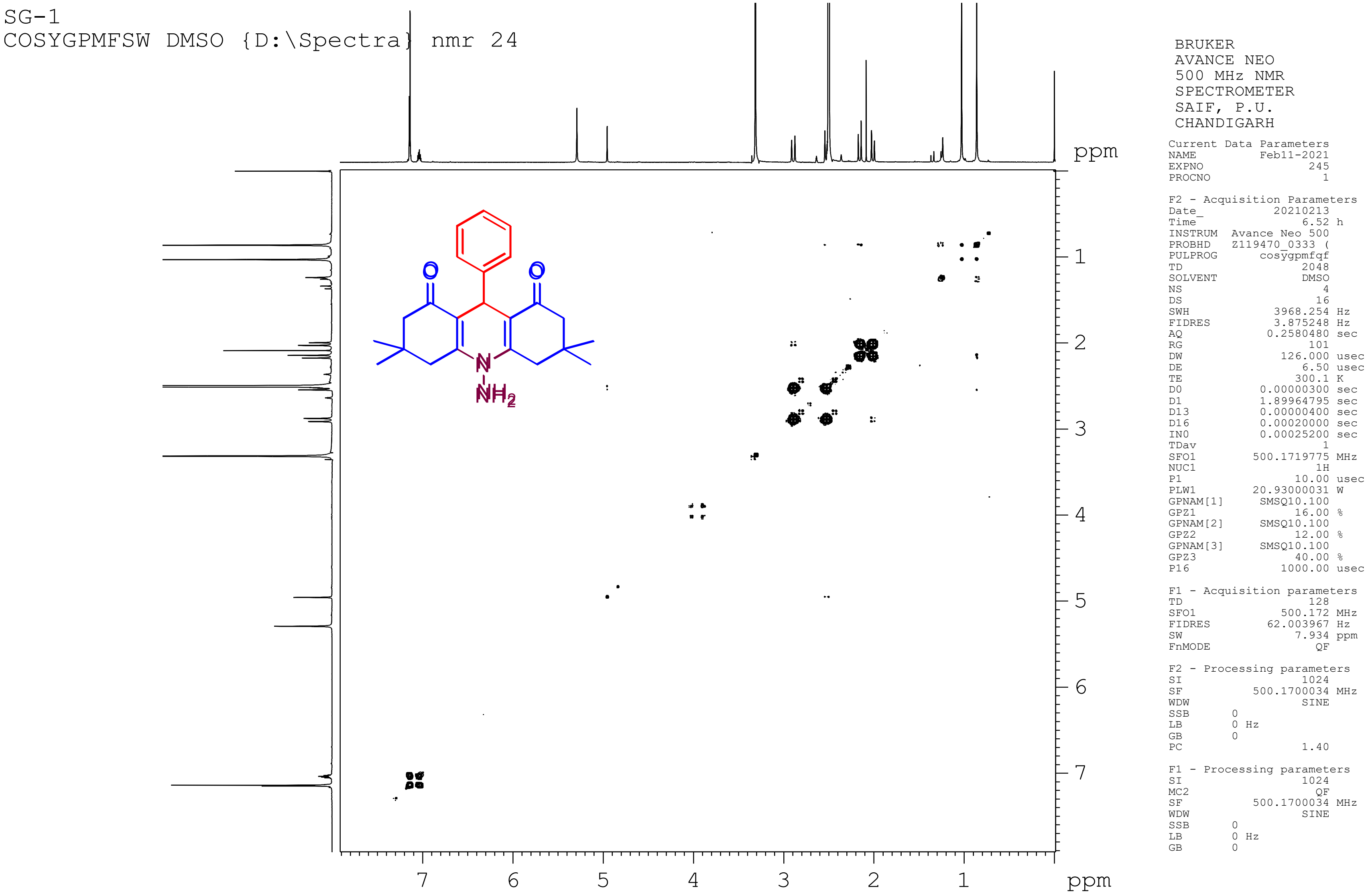

Figure S11. COSY expanded spectrum of *10-amino-3,3,6,6-tetramethyl-9-phenyl-3,4,6,7,9,10-hexahydroacridine-1,8(2H,5H)-dione* ***(4a)***.

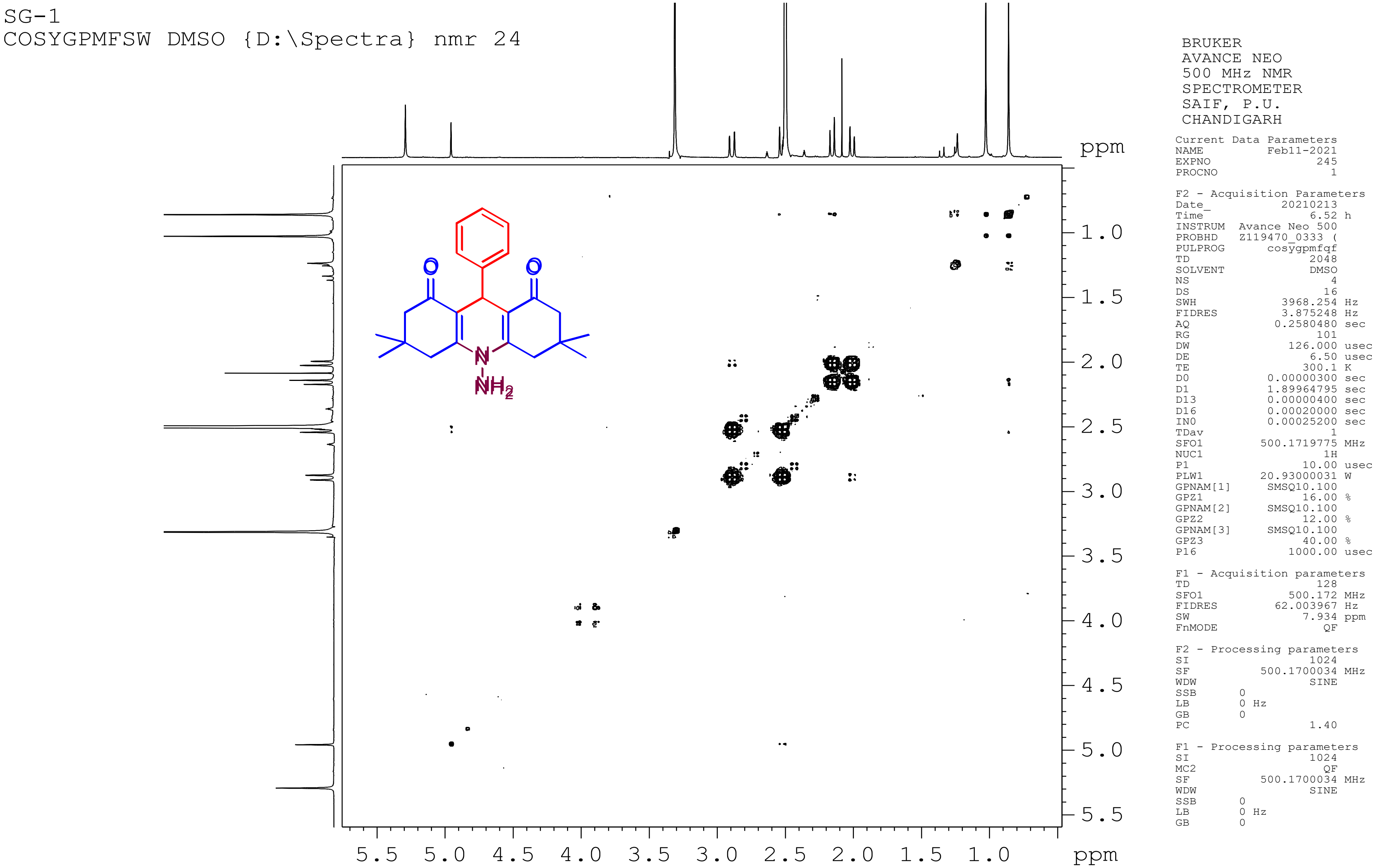

Figure S12. COSY expanded spectrum of *10-amino-3,3,6,6-tetramethyl-9-phenyl-3,4,6,7,9,10-hexahydroacridine-1,8(2H,5H)-dione (4a)*.

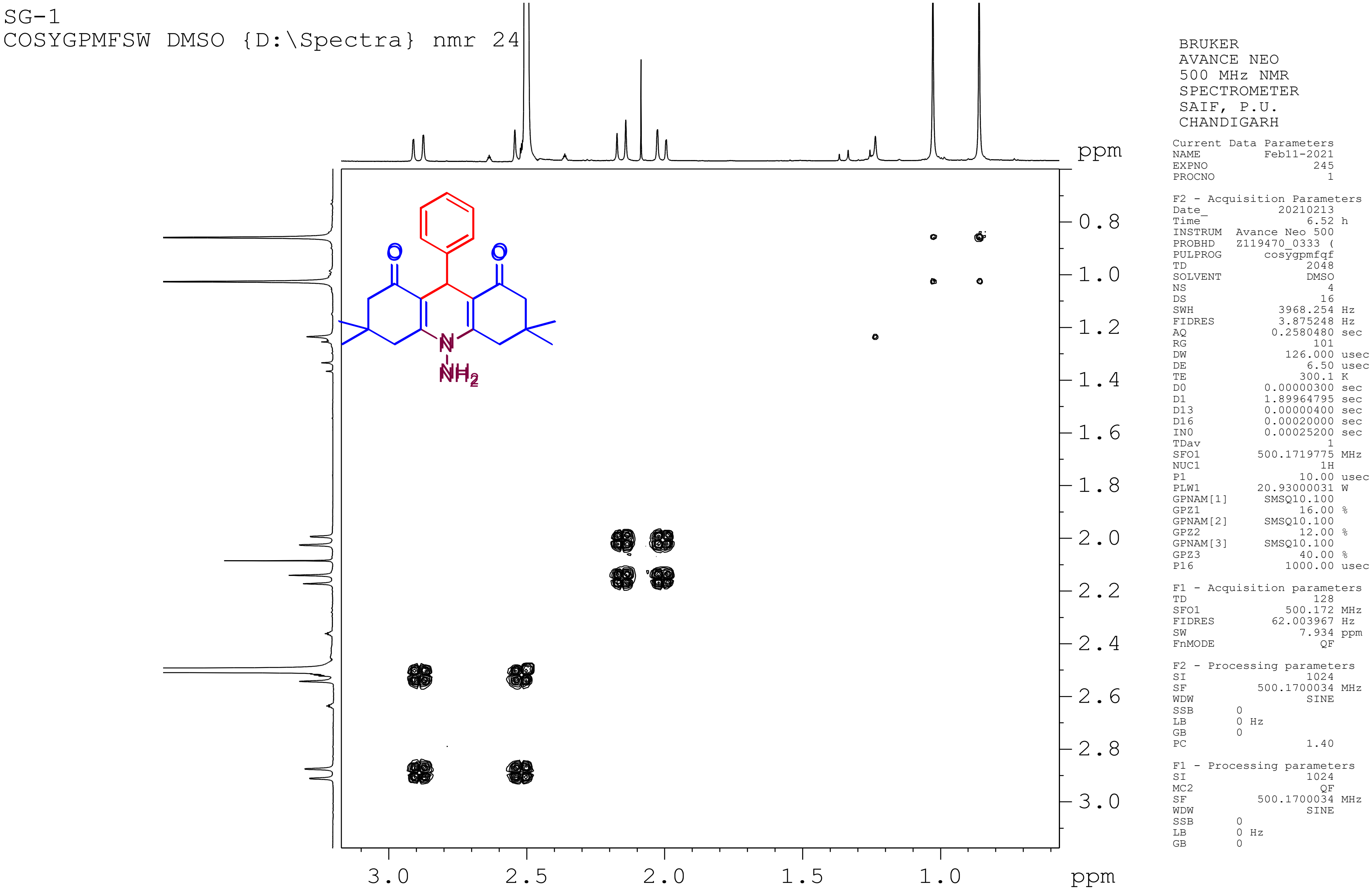

Figure S13. COSY expanded spectrum of *10-amino-3,3,6,6-tetramethyl-9-phenyl-3,4,6,7,9,10-hexahydroacridine-1,8(2H,5H)-dione (4a)*.

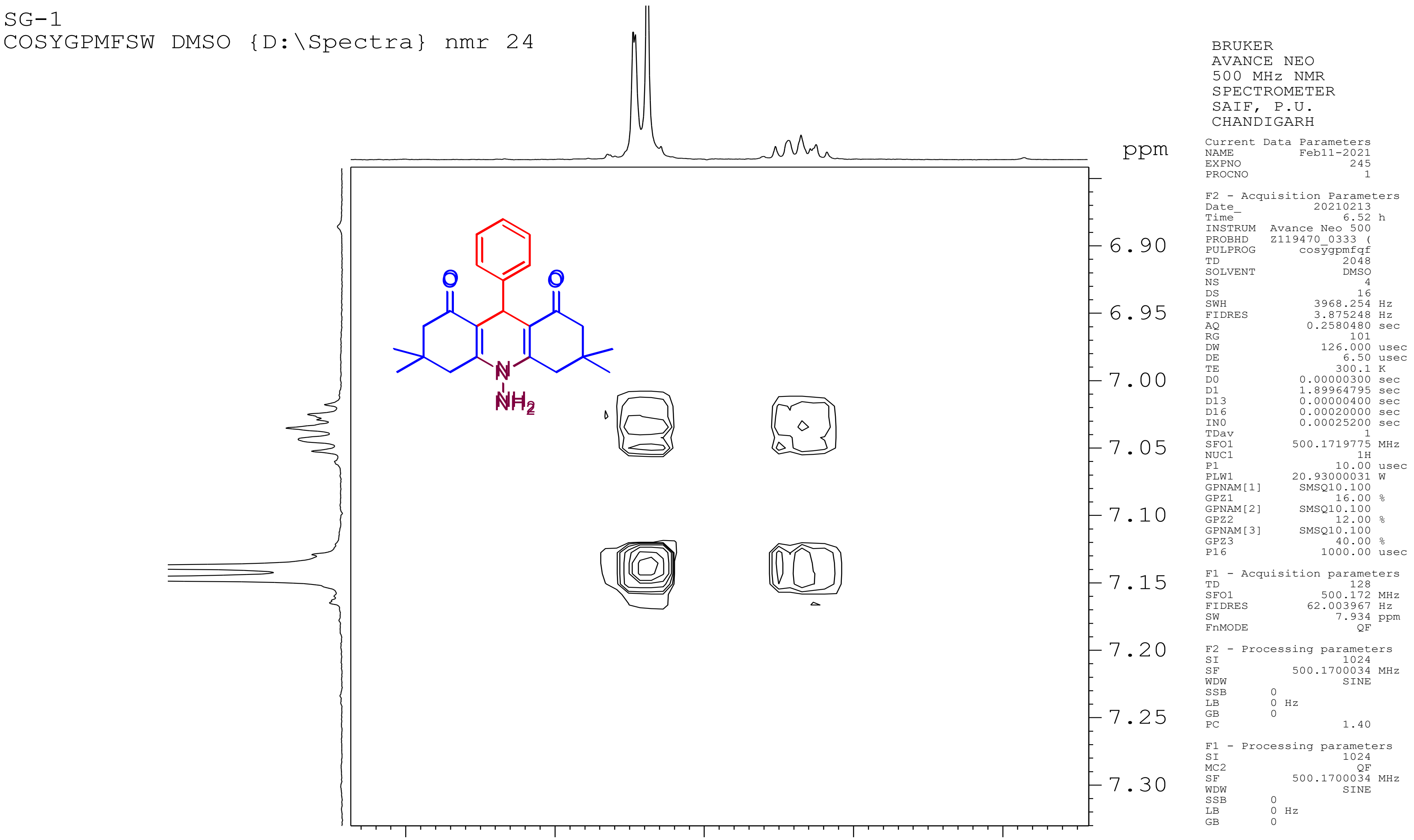

Figure S14. COSY expanded spectrum of *10-amino-3,3,6,6-tetramethyl-9-phenyl-3,4,6,7,9,10-hexahydroacridine-1,8(2H,5H)-dione **(4a)***.

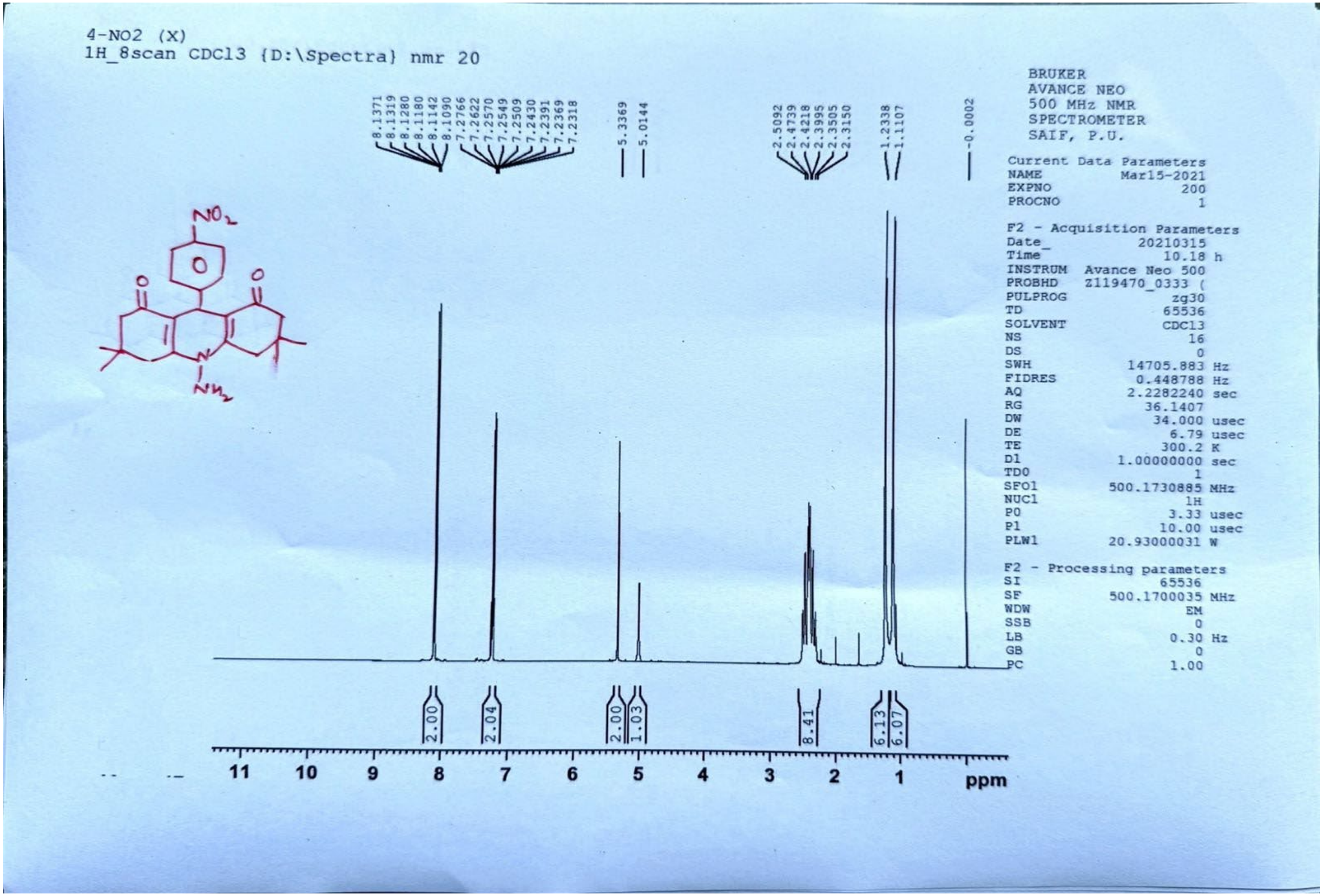

Figure S15. [1]**H NMR** spectrum of ***10-amino-3,3,6,6-tetramethyl-9-(4-nitrophenyl)-3,4,6,7-tetrahydroacridine-1,8(2H,5 H,9H,10H)-dione (4b).***

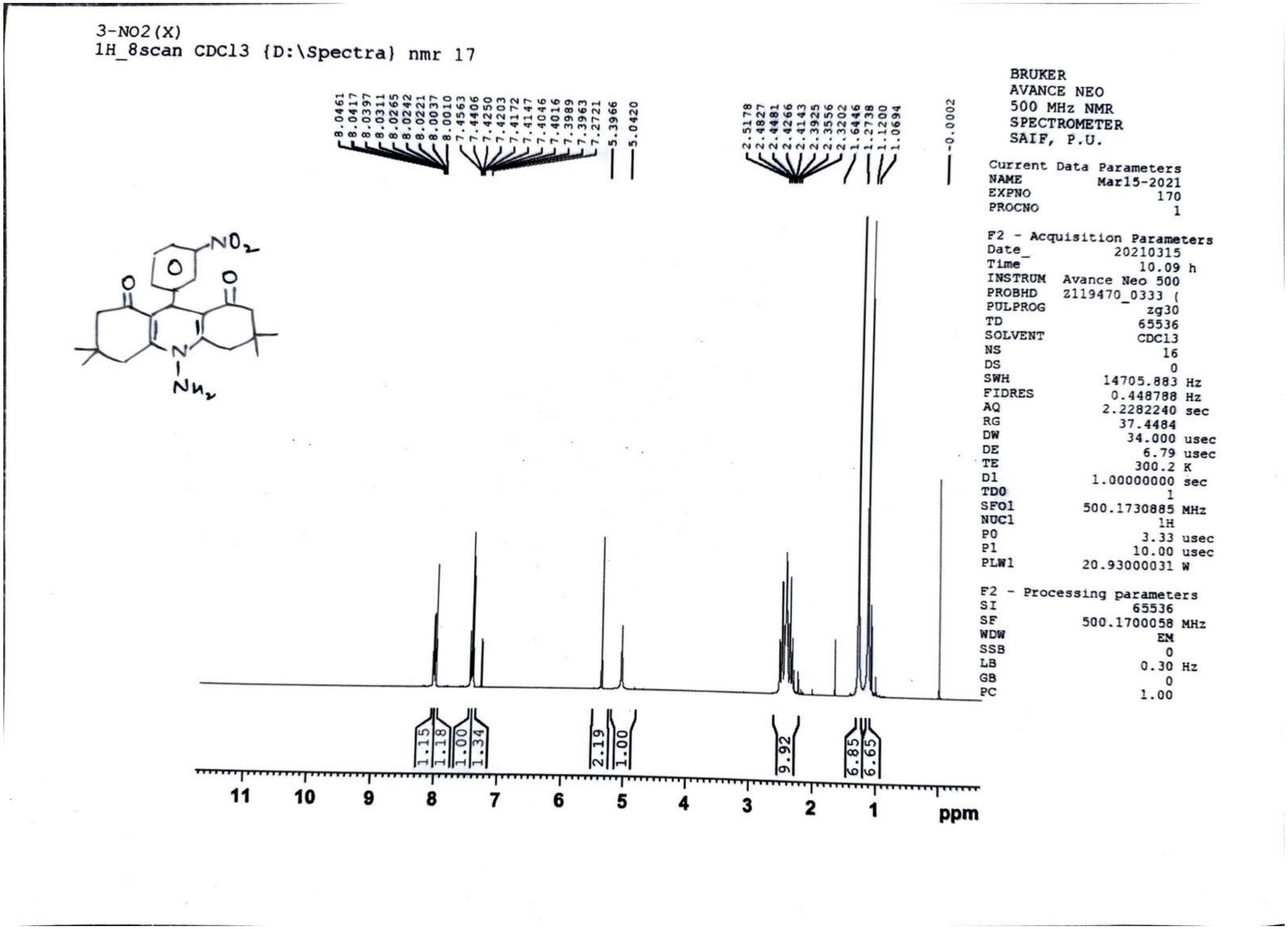

Figure S16. **^{1}H NMR** spectrum of ***10-amino-3,3,6,6-tetramethyl-9-(3-nitrophenyl)-3,4,6,7-tetrahydroacridine-1,8(2H,5H,9H,10H)-dione (4c)***.

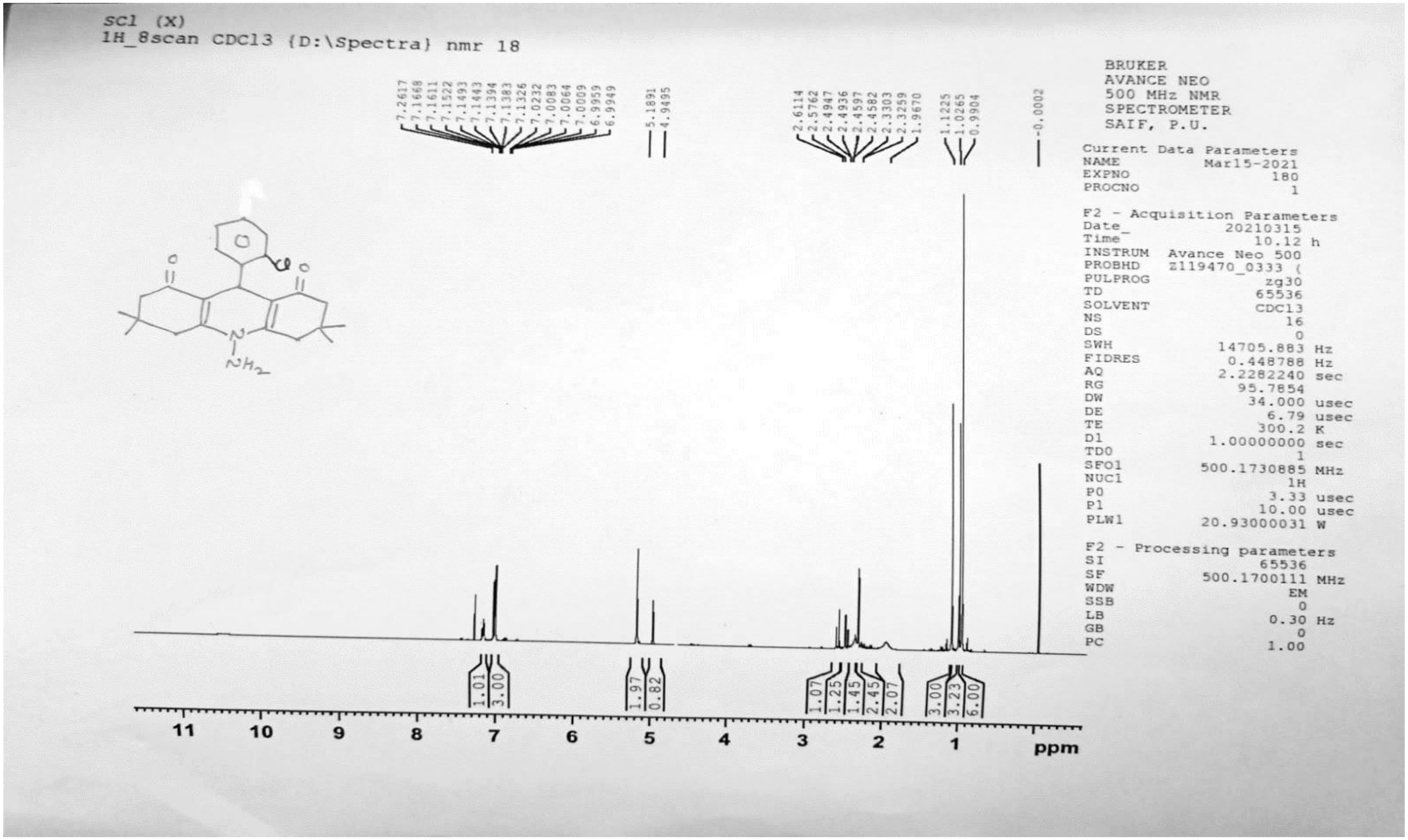

Figure S17. ^{1}**H NMR** spectrum of ***10-amino-9-(2-chlorophenyl)-3,3,6,6-tetramethyl-3,4,6,7-tetrahydroacridine-1,8(2H, 5H,9H,10H)-dione (4f)***.

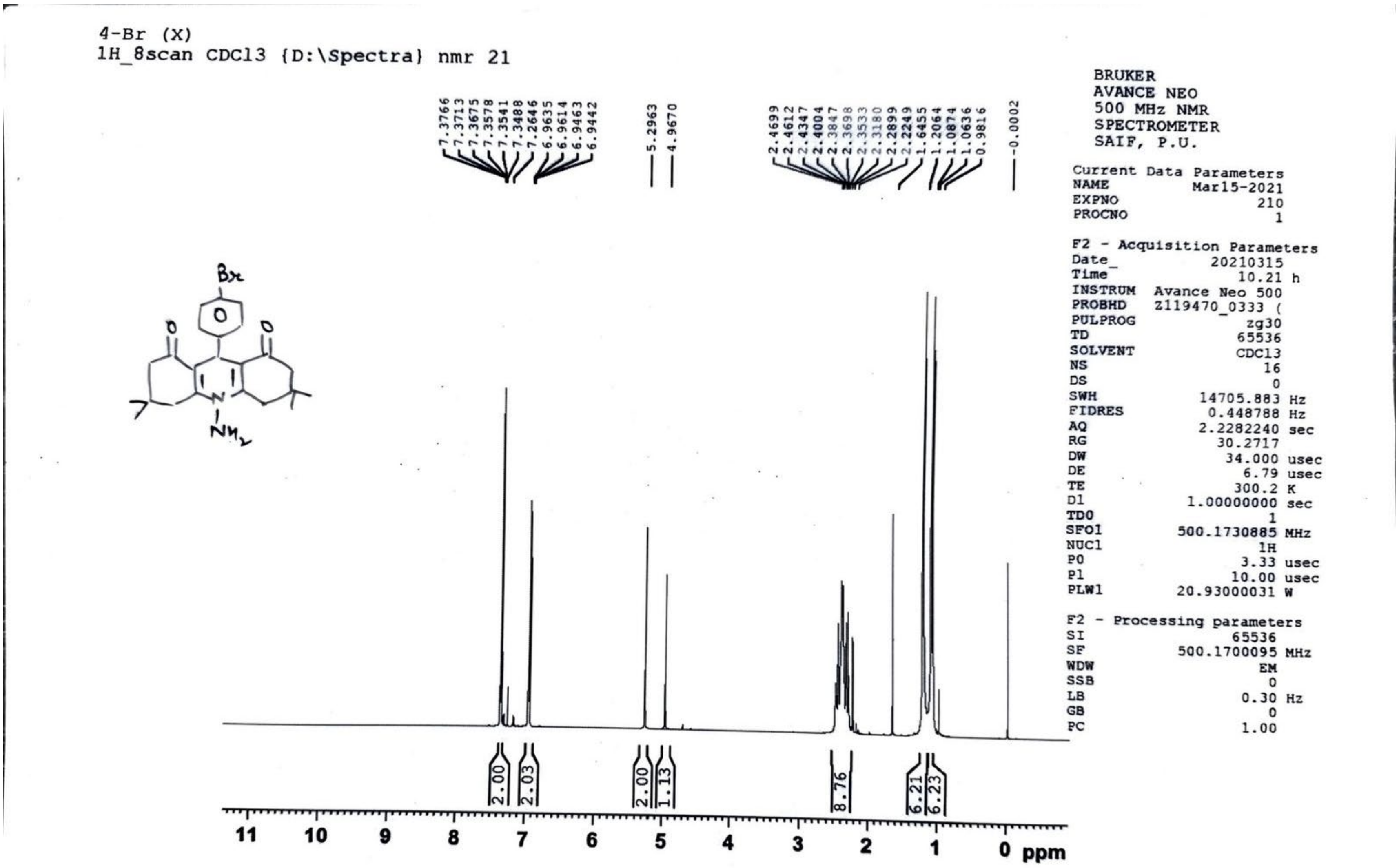

Figure S18. ^{1}**H NMR** spectrum of ***10-amino-9-(4-bromophenyl)-3,3,6,6-tetramethyl-3,4,6,7-tetrahydroacridine-1,8(2H, 5H,9H,10H)-dione (4g)***

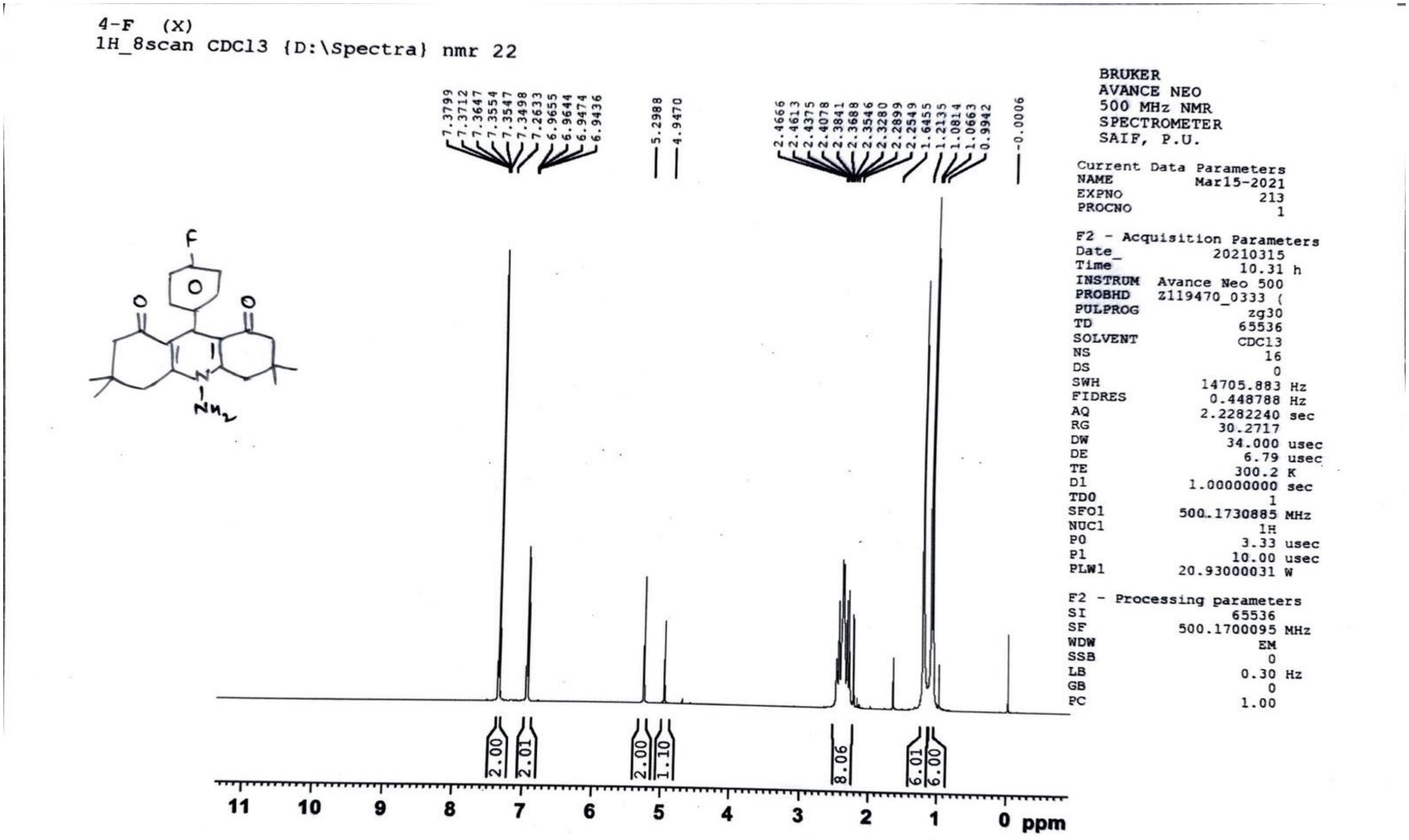

Figure S19. **^{1}H NMR** spectrum of ***10-amino-9-(4-florophenyl)-3,3,6,6-tetramethyl-3,4,6,7-tetrahydroacridine-1,8(2H, 5H,9H,10H)-dione (4h)***

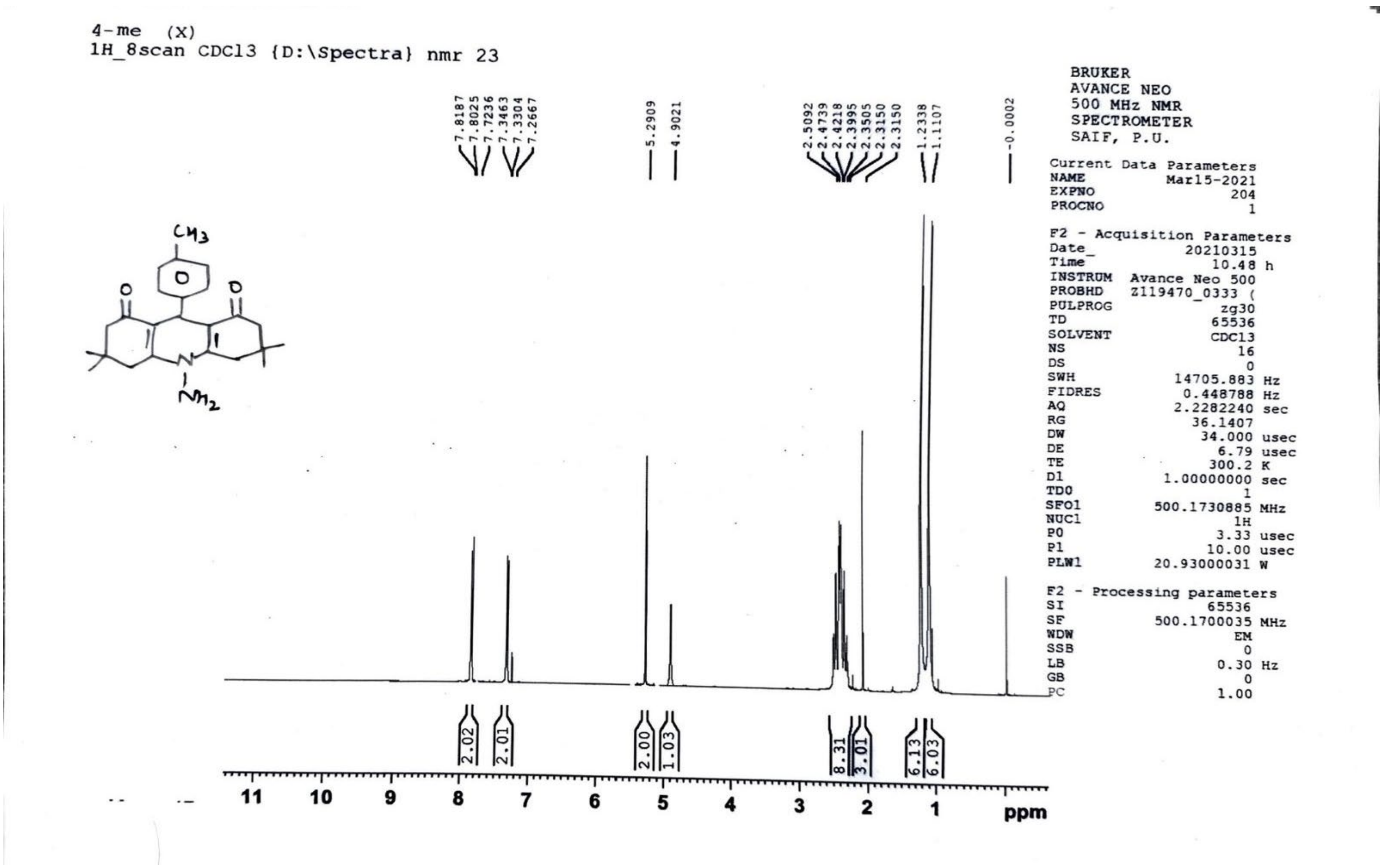

Figure S20. [1]**H NMR** spectrum of ***10-amino-3,3,6,6-tetramethyl-9-p-tolyl-3,4,6,7-tetrahydroacridine-1,8(2H,5H,9H,10 H)-dione (4l).***

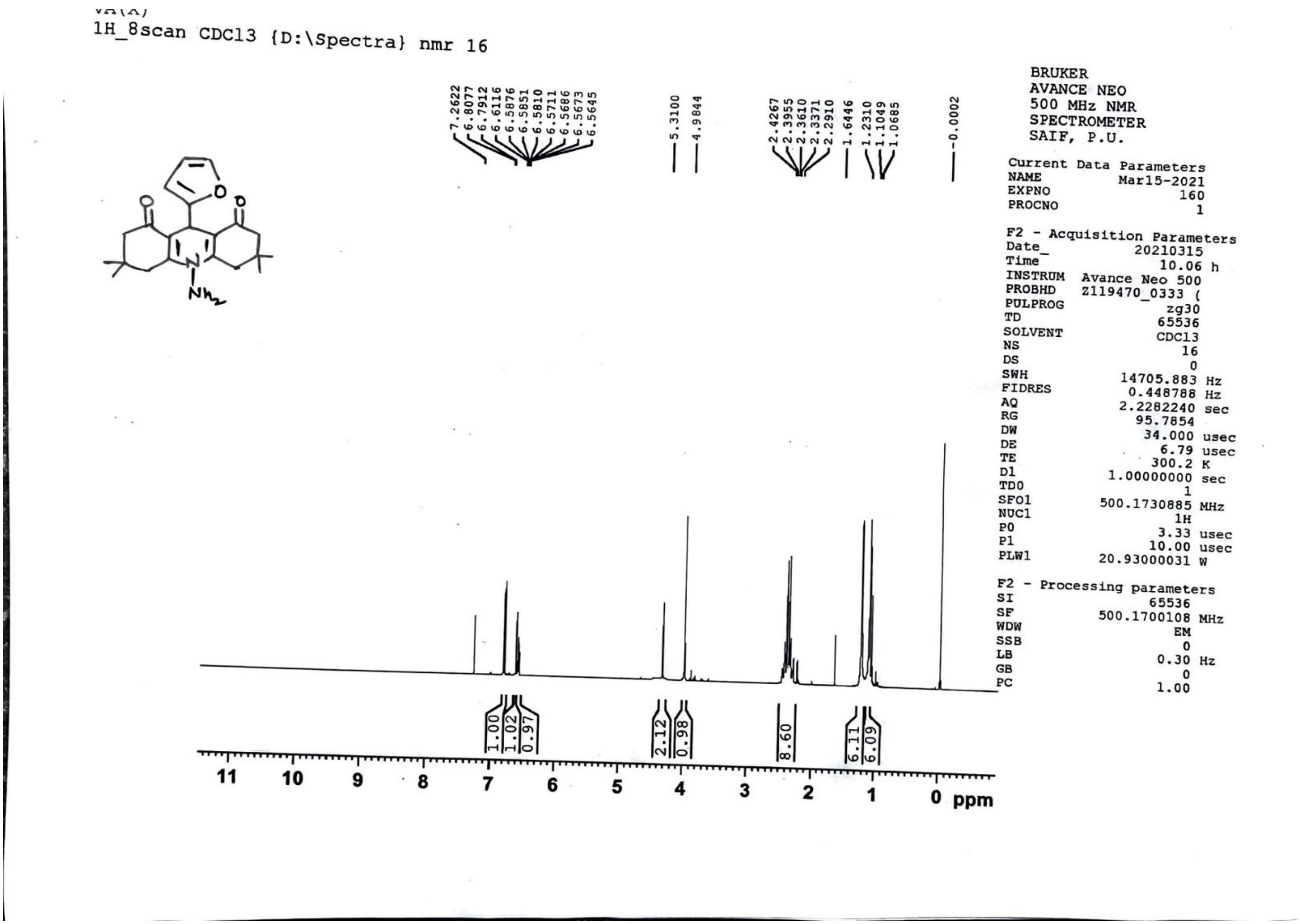

Figure S21. ^{1}H **NMR** spectrum of ***10-amino-9-(furan-2-yl)-3,3,6,6-tetramethyl-3,4,6,7-tetrahydroacridine-1,8(2H,5H, 9H,10H)-dione (4o)***

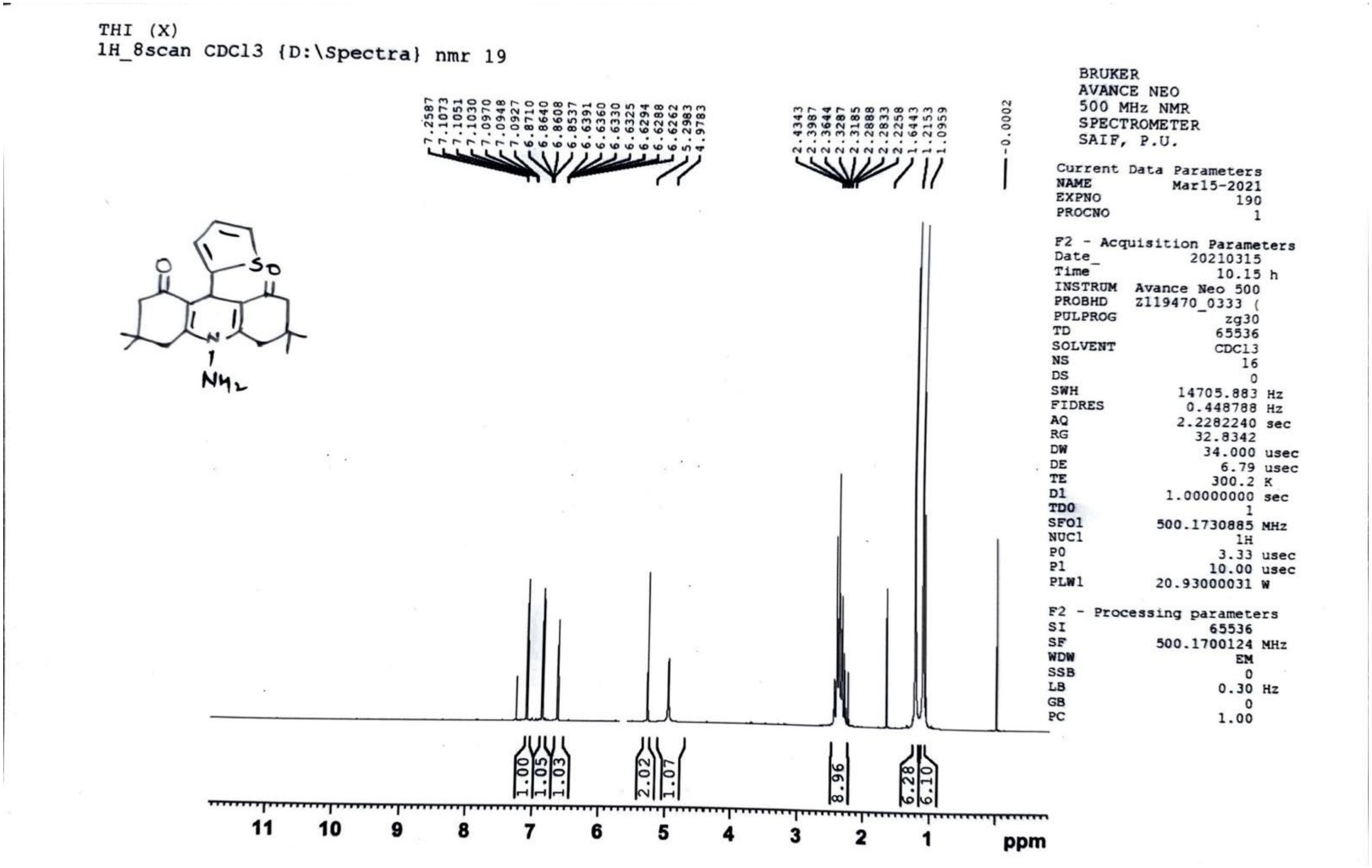

Figure S22. ^{1}**H NMR** spectrum of ***10-amino-3,3,6,6-tetramethyl-9-(thiophen-2-yl)-3,4,6,7-tetrahydroacridine-1,8(2H,5 H,9H,10H)-dione (4p).***
